# Selective Mutism and Its Relations to Social Anxiety Disorder and Autism Spectrum Disorder

**DOI:** 10.1007/s10567-020-00342-0

**Published:** 2021-01-19

**Authors:** Peter Muris, Thomas H. Ollendick

**Affiliations:** 1 Department of Clinical Psychological Science, Faculty of Psychology and Neuroscience, Maastricht University, P.O. Box 616, 6200 MD Maastricht, The Netherlands; 2 Stellenbosch University, Stellenbosch, South Africa; 3 Virginia Polytechnic Institute and State University, Blacksburg, USA; 4 Roehampton University, London, England

**Keywords:** Selective mutism (SM), Social anxiety disorder (SAD), Autism spectrum disorder (ASD)

## Abstract

In current classification systems, selective mutism (SM) is included in the broad anxiety disorders category. Indeed, there is abundant evidence showing that anxiety, and social anxiety in particular, is a prominent feature of SM. In this article, we point out that autism spectrum problems in addition to anxiety problems are sometimes also implicated in SM. To build our case, we summarize evidence showing that SM, social anxiety disorder (SAD), and autism spectrum disorder (ASD) are allied clinical conditions and share communalities in the realm of social difficulties. Following this, we address the role of a prototypical class of ASD symptoms, restricted and repetitive behaviors and interests (RRBIs), which are hypothesized to play a special role in the preservation and exacerbation of social difficulties. We then substantiate our point that SM is sometimes more than an anxiety disorder by addressing its special link with ASD in more detail. Finally, we close by noting that the possible involvement of ASD in SM has a number of consequences for clinical practice with regard to its classification, assessment, and treatment of children with SM and highlight a number of directions for future research.

## Introduction

### Two Cases

Ibi is a 10-year-old boy of an Algerian immigrant family who was born in the Netherlands. His parents speak a mix of Arabic (father), Dutch and French (mother), and at home the boy is perfectly able to express himself in all three languages. In fact, most of the time, he is quite noisy, continuously chatting to his younger brother while they play. However, the picture at school is totally different: Ibi does not utter a single word during the whole day. When entering school in the morning, the teacher greets him in a friendly manner, but Ibi only nods in a grumpy way and does not respond verbally. In class, he follows the instructions of the teacher and conducts all the tasks as long as they are non-verbal in nature. Ibi is never observed talking to his peers; in general, he is quite shy and withdrawn at school, but sometimes he uses gestures to clarify his intentions and needs. For instance, during the lunch break, when the class is playing dodgeball, Ibi is eager to join the game. However, he is not shouting like the other children but constantly waving his arms, trying to prompt his teammates to throw the ball to him. He seems frustrated when they are not responsive to this request and sometimes roughly pushes other children aside.

Leo is the 11-year-old son of a native Dutch couple who has been referred by the neurologist who could not find a somatic cause for the boy’s recurrent severe headaches. The doctor in the hospital noted that Leo was tense and nervous and remained totally silent during the two consultations that he had with the boy and his parents. The parents confirm that Leo can remain totally mute when meeting unfamiliar people. He prefers to avoid or withdraw from such situations, but when unable to do so he does not speak at all and at times even displays strange, regressive behaviors. Leo then assumes a shrunken position, closes his eyes, and sucks on his thumb. The teacher at school has also experienced this once when Leo had to provide a speech in front of the class. She reports that Leo sometimes exchanges a few words in private with her before or after school and that he lately whispers silently to the boy sitting next to him, but he has never spoken out loud in class.

Both boys were diagnosed with selective mutism (SM), a psychiatric condition typically occurring during childhood that is characterized by a consistent failure to speak in specific social situations in which there is an expectation for speaking (e.g., at school) despite displaying the capability to speak normally in other situations (like at home; American Psychiatric Association [APA] 2013). Ibi and Leo were referred to an outpatient facility specialized in the treatment of childhood anxiety disorders. These referrals seemed appropriate given the prominent role of anxiety, and in particular social anxiety, in the clinical picture of this psychiatric disorder (e.g., Driessen et al. 2020; Muris and Ollendick 2015), which has resulted in its allocation to the category of anxiety disorders in current versions of classification systems including the Diagnostic and Statistical Manual of Mental Disorders (DSM-5; APA 2013) and the International Classification of Diseases (ICD-11; World Health Organization [WHO] 2018). A standardized questionnaire taken at the clinic confirmed the presence of anxiety problems and revealed clinically elevated scores, not only for social anxiety (both boys), but also for a number of other anxiety problems such as separation anxiety (Ibi), generalized anxiety (Leo), and specific phobias (both boys).

However, during additional assessment it became clear that the psychopathology of both boys was far more complicated. A detailed developmental anamnesis revealed social peculiarities from the beginning of life for both boys, including abnormalities in responsive social behaviors such as smiling and eye contact, a lack of interest in other children, reduced sharing of emotions and intentions, and recurrent problems with peers. Further, it was noted that Ibi and Leo had an insistence on sameness and were easily upset by small changes in daily routines and unexpected events. Both of them had at least one special preoccupation (Ibi: Pokémon; Leo: numbers), regularly displayed repetitive behavior (Ibi: in the past hand flapping, nowadays typical hand movements; Leo: lining up toys), and at times were difficult to handle because of rigidity in their behavior.^1^ Given these persistent deficits in social communication and social interaction across various contexts and the presence of rigid, restricted, repetitive patterns of behavior, interests, and activities, a diagnosis of autism spectrum disorder (ASD) also seemed justified (APA 2013). However, this comorbidity is formally not permitted because classification systems such as DSM-5 and ICD-10 indicate the presence of ASD as an exclusion criterion for defining SM.

The cases of Ibi and Leo nicely illustrate what we view as the clinical trinity of abnormal social behavior, which consists of SM, SAD, and ASD. The key point that we want to make in this review is that SM, at least in some children diagnosed with this condition, is more than just an anxiety disorder and SAD in particular, but that ASD-related features be present as well (Holka-Pokorska et al. 2018). The structure of this review is as follows. First, we will describe the diagnostic features of SM, the prevalence of this disorder, and its development and course. Then, we will look at relations between SM and SAD and between SM and ASD. Next, we discuss the commonalities and differences in social difficulties of the three disorders in some detail. Following this, we address the role of a prototypical class of ASD symptoms, restricted and repetitive behaviors and interests (RRBIs), which we hypothesize to play a special role in the preservation and exacerbation of social difficulties. Subsequently, we will substantiate our point that SM is sometimes more than an anxiety disorder by addressing its special link with ASD. Finally, we will discuss implications for the classification, assessment, and treatment of children and adolescents displaying SM and co-occurring social difficulties as well as point out directions for future research on this disorder.

## Selective Mutism (SM)

The prominent feature of SM is that children with this condition do not initiate speech or do not respond when spoken to by others in specific situations (e.g., in school or when meeting unfamiliar adults or peers), whereas they are perfectly able to speak in other settings (e.g., at home with their parents and siblings, with other family members such as grandparents, and with close friends). To make the diagnosis, the DSM-5 (APA 2013) requires that the failure to speak in certain situations is not attributable to a lack of knowledge of, or discomfort with, the spoken language required in these situations. Further, the duration of the disturbance is at least one month (and should not be limited to the first 4 weeks in school), and the problem should interfere with the educational (or occupational) achievement or social communication of the young person. Finally, the disturbance is not better explained by a communication disorder (e.g., childhood-onset fluency disorder) and does not occur exclusively during the course of ASD (which is a point that will be addressed in more detail later), schizophrenia, or another psychotic disorder.

Although not explicitly indicated in the diagnostic criteria, anxiety, and in particular social anxiety, is a common feature of children with SM. However, other problems such as oppositional behavior and (mild) language and communication problems may also be present. In an interesting study by Cohan et al. (2008), symptoms of 130 children aged 5 to 12 years who were diagnosed with SM were examined by means of latent class analysis in order to identify subgroups of the disorder. A three-class model was found that provided the best fit for the data. Class 1 represented 44.6% of the total sample and consisted of children with clinically elevated scores for social anxiety and borderline clinical scores of behavioral problems, which was labelled as the anxious-mildly oppositional group. Class 2 contained 43.1% of the sample and included children with clinically significant scores for social anxiety and borderline clinical levels of language and speech problems. This class was labelled as the anxious-communication delayed group. Class 3 consisted of 12.3% of the sample and contained children who only had clinically elevated anxiety scores and were labelled as the exclusively anxious group. These findings indicate that in most children with SM anxiety is a prominent feature, although in a substantial proportion other problems are present as well, making it a rather heterogeneous disorder.

There is general consensus that SM is a rare psychiatric condition, although epidemiological studies on its occurrence are relatively scarce. Two studies assessed the prevalence of the disorder in clinical populations. In one study, Carlson et al. (1994) asked psychiatrists working in child and adolescent mental health settings whether they had ever diagnosed and/or treated a young person with SM. Sixty-five per cent of the 308 participating psychiatrists responded positively to this question and a total of 670 children with the disorder were identified. Based on an estimation of the total number of patients that the psychiatrists had seen during their career, the overall prevalence of SM was estimated at 0.11%. In another investigation, Steinhausen and Juzi (1996) directly searched the case files of two child and adolescent psychiatry clinics in Zurich, Switzerland and Berlin, Germany, which yielded prevalence rates of 0.44% and 0.54%, respectively. Other researchers have examined the prevalence of SM in school-based samples by first asking teachers to identify extremely shy and reticent children in their classrooms, after which a psychological and/or psychiatric assessment was conducted to establish whether these children met the diagnostic criteria of the disorder. Using this approach, prevalence rates varied between 0.03 and 1.89% (Bergman et al. 2002: 0.71%; Elizur and Perednik 2003: 0.76%; Karakaya et al. 2008: 0.03%; Kumpulainen et al. 1998: 1.89%; Sharkey and McNicholas 2012: 0.18%), depending on sample characteristics, informants and assessment instruments, and exact diagnostic criteria employed to define the disorder.

SM is typically an early-onset disorder, starting usually before the age of 5 years and often becoming a focus of clinical attention when children enter school (Kristensen 2000). Symptoms tend to decrease as children become older. For example, in the study by Bergman et al. (2002), 12 kindergarten, first and second grade children with SM were followed for a 6-month period, and it was noted that according to the teachers the frequency of the speaking behavior significantly increased while social anxiety substantially decreased over the 6 months. However, scores of the children with SM remained higher than the normative range. Moreover, findings from long-term follow-up studies show that although the prototypical muteness gradually diminishes in most cases, social and communication problems tend to continue into adolescence and even adulthood (Remschmidt et al. 2001; Steinhausen et al. 2006). Most research has shown that SM, just like most other members of the anxiety disorders family, is more common among girls than boys (e.g., Elizur and Perednik 2003; Kumpulainen et al. 1998; Sharkey and McNicholas 2012), but some studies do not report such a gender difference (e.g., Bergman et al., 2002). Moreover, another investigation even found a preponderance of boys over girls (Karakaya et al., 2008).

## Relations Between SM and SAD/ASD

In this section, we will summarize existing evidence on the link between SM and two other types of psychopathology that are also characterized by social difficulties, namely SAD and ASD. The primary focus will be on comorbidity data; however, if such data are not available, we will describe other findings that support the notion that SM is related in meaningful ways to both SAD and ASD.

### SM and SAD

The key feature of SAD is a marked and disproportionate fear or anxiety about one or more social situations involving exposure to possible critical evaluation by others (APA 2013). In children, the fear and anxiety must also occur in peer settings and not just during interactions with adults. Examples include typical school situations such as answering a question of the teacher or writing on the blackboard as well as extracurricular activities such as performing in front of others during musical events or sports, starting or joining in on a conversation, and eating in a restaurant or other public places (Beidel et al. 1999). A particularly strong link has been noted between SM and SAD, and the evidence for this relationship comes from at least four lines of research.

The first line of research consists of studies in which (semi)structured clinical interviews were administered to study comorbidity patterns of SM with other anxiety disorders. This research has indicated that up to 80% of the children with SM also meet the diagnostic criteria for another anxiety disorder, of which SAD is the most frequently established comorbid condition. For example, in a recent meta-analysis comprising a total sample of 837 children with SM, Driessen et al. (2020) found that, on average, 69% of the children were also diagnosed with SAD. Admittedly, this percentage was quite variable across the 22 included studies, with multiple investigations reporting percentages of 100%, but also one study reporting a percentage as low as 0% (Nowakowski et al. 2011). Note, however, that in all these studies SAD was classified as a categorical disorder rather than as a dimensional phenomenon, and that it thus remains possible that even in the absence of a formal diagnosis subclinical levels of social anxiety symptoms may still have been present.

The second research line indeed relies more on the notion that psychopathological phenomena should be viewed on a continuum and explores to what extent children with SM display varying levels of social anxiety and several other types of symptoms. The aforementioned study by Cohan et al. (2008) is a good example of such an approach, and in general this type of research has shown that although SM is associated with a heterogeneous set of symptoms, social anxiety appears to be a prominent feature that is present in almost all children with this psychiatric condition (see also Diliberto and Kearney 2016, 2018; Schwenck et al. 2019).

A third line of inquiry, which surprisingly has only been undertaken quite recently, has used the method of directly investigating the content of the fears and fear-related cognitions of children with SM. Vogel et al. (2019), for instance, used an online survey containing an open-ended question asking 65 children with SM (aged 8–18 years) to specify the content of their fear in situations in which they were expected but not able to speak. Further, children with SM but also children with SAD (*n* = 18) and typically developing children (*n* = 51) were asked to complete a questionnaire containing a set of fear-related cognitions that might occur in speech-demanding situations. Qualitative content analysis of the responses that children with SM gave to the open-ended question revealed that fears belonging conceptually to SAD were most prominent (59%). Other fear categories reported by Vogel et al. (2019) were fear of making mistakes (e.g., fear of giving a wrong answer), language-related fears (e.g., fear of not finding the right words, fear of poor articulation), and voice-related fears (i.e., fear that one’s voice sounds funny or odd), but one could argue that these fears—although somewhat atypical in content—are strongly indicative of social anxiety as well. On the self-constructed questionnaire, children with SM displayed equally high levels of negative fear cognitions as children with SAD, with both clinical groups showing significantly higher scores than the group of typically developing children.

A fourth and final line of research is concerned with the temperament typology of behavioral inhibition (BI), which has been defined as the tendency to react with shyness, distress, and withdrawal in response to novel and challenging situations (Kagan 1994). Various studies have shown that BI is a consistent correlate of SAD (e.g., Ollendick and Benoit 2012), and most importantly a meta-analysis of Clauss and Blackford (2012) revealed that this temperament factor is associated with a greater than sevenfold increase in the risk for developing this anxiety disorder. Interestingly, a number of recent studies have investigated this temperament factor in children with SM. For example, Gensthaler et al. (2016a) compared levels of BI in children aged 3 to 18 years with SM, SAD, or other internalizing behaviors, and healthy controls using the parent-rated Retrospective Infant Behavioral Inhibition (RIBI) questionnaire (Gensthaler et al. 2012). The results indicated that children with SM and SAD were reported to be more inhibited when they were infants and toddlers than children with other internalizing behaviors and healthy controls. Further, it was found that children with SM even showed higher total BI scores and in particular on the RIBI subscale referring to shyness than children with SAD. In another study by Muris et al. (2016), 57 non-clinical children aged 3 to 6 years performed two speech tasks to assess their absolute amount of spoken words, while their parents completed a set of questionnaires for measuring children’s levels of SM and social anxiety symptoms as well as BI. Significant associations were noted among all these variables, but the correlation between BI and SM symptoms was particularly robust (*r* = 0.64), and it was also found that this temperament characteristic was the best predictor of the number of spoken words during the standardized speech tasks. Altogether, these findings indicate that BI, which has been established as an important risk factor for SAD, is also implicated in SM, and this of course underlines the connection between both disorders.

In conclusion, there is considerable evidence from various types of research for the strong link between SM and social anxiety. That is, children with SM are often diagnosed with comorbid SAD, display high levels of social anxiety symptoms, have fears and fear cognitions that are highly comparable in terms of content and severity, and share a similar temperamental vulnerability (i.e., BI) as those reported by young people with SAD (Chavira et al. 2007). The relation between SM and SAD is so intimate that it has been argued by some scholars that the two are one and the same disorder (Bögels et al. 2010). Some advocates of this idea have suggested that SM should be considered as a more extreme variant of SAD (e.g., Black and Uhde 1992), while others have put forward that SM can best be seen as an early developmental manifestation of SAD (e.g., Bergman et al. 2002).

### SM and ASD

ASD is a neurodevelopmental disorder characterized by (1) persistent deficits in the reciprocal social communication and social interaction, and (2) restricted, repetitive patterns of behavior and interests (RRBIs; APA 2013). ASD is currently perceived as a spectrum disorder, which means that we no longer classify children with this psychiatric condition in separate diagnostic categories such as autistic disorder, Asperger’s disorder, childhood disintegrative disorder, Rett’s disorder, and pervasive developmental disorder not otherwise specified (APA 1994), but rather qualify the severity of the deficits and problems on a three-level scale ranging from mild (requiring support) to severe (requiring very substantial support; APA 2013).

Research on the link between SM and ASD is sparse. The main reason seems to be the artificial boundary that classification systems have placed between both disorders. For example, in DSM-5 (APA 2013) it is noted that the failure to speak as the key feature of SM should not exclusively occur during the course of ASD, which in practice is quite difficult to determine given that the latter disorder is currently perceived as a dimension, which implies that the demarcation between SM and ASD has become more blurred. Ever since SM was included in the psychiatric classification systems, researchers (and clinicians; see Simms 2017; Snyder et al. 2008) have struggled with the ASD-related exclusion criterion and tended to ignore the co-occurrence of both disorders. For example, in the study by Anderson and Thomsen (1998) who described the sociodemographic and clinical features of 37 clinically referred cases with SM, it was found that nearly half of the children showed significant developmental problems. Only a substantial minority of them (8.1%) met the criteria of Asperger’s syndrome, but it is important to note that children with more severe ASD had already been excluded from the sample because these problems were considered as too pervasive to be relevant in relation to SM. Another investigation by Kristensen (2000) adopted a similar approach and noted that 7.4% of the children with SM fulfilled the criteria for Asperger’s disorder, whereas on a teacher questionnaire for measuring symptoms of Asperger’s syndrome a considerably higher percentage (25.5%) showed elevated scores.

In a population-based study of 2973 Swedish school children aged 7 to 15 years, Kopp and Gillberg (1997) detected five children with SM using a teacher-based screening questionnaire. Due to the procedural set-up of the study, only two of these five children were subjected to an in-depth neuropsychiatric examination and it appeared that one of them had clear signs of ASD. Besides the absence of speech in the presence of strangers, this child also displayed severe empathy problems, showed little social awareness of others, had no friends, and exhibited a circumscribed interest involving rote memory learning. The researchers were cautious enough to interpret the presence of ASD in SM as a chance finding, but also noted that both disorders could be associated in a meaningful manner but that this can only be ascertained when classification systems allow the two conditions to be diagnosed in the same individual.

Cholemkery et al. (2014) explored the extent to which SM (and SAD) and ASD can be differentiated in terms of impairments in social interactions. Parents of 6- to 18-year-old children with SM (*n* = 43), SAD (*n* = 38), and ASD (*n* = 60) as well as typically developing children (*n* = 42) completed the Social Responsiveness Scale (SRS; Constantino and Gruber 2005), which measures five symptom domains indicative of ASD, namely social awareness, social cognition, social communication, social motivation, and autistic mannerisms. The results showed that children with ASD, SM, and SAD displayed higher SRS scores than the typically developing children. Further, there was considerable overlap among the three disorders, although children with ASD generally showed the highest levels of social interaction impairments. However, on two domains (i.e., social communication and social motivation) children with SM also displayed elevated scores (as compared to children with SAD), which suggests that they were relatively high on the autism spectrum and thus showed at least some signs of this neurodevelopmental disorder.

More recently, Steffenburg et al. (2018) conducted a study in which the comorbidity between SM and ASD was investigated in a systematic way. The medical records of 97 clinically referred children between 4 and 18 years of age were analyzed in detail to retrospectively verify the presence of autism spectrum problems, thereby forgoing the exclusion criterion prescribed by classification systems such as DSM and ICD. During the initial intake assessment, children and parents had been subjected to an extensive diagnostic procedure that also included specific instruments for measuring symptoms of ASD. All children had received SM as the primary diagnosis on the basis of the referral question and which treatment had been initiated, but the additional post-hoc analysis by Steffenburg et al. revealed that 61 (62.9%) of the children with SM could also be diagnosed with ASD. More precisely, in DSM-IV (APA 1994) terminology, 28 (28.9%) of them had autistic disorder, 4 (4.1%) had Asperger’s syndrome, and 29 (29.9%) had pervasive developmental disorder-not otherwise specified. Moreover, a substantial number of the children who did not receive a formal ASD diagnosis (*n* = 19, 19.6%) still displayed “subclinical” autistic features, which means that only 17 (17.5%) of the children with SM showed no overt signs of ASD. Admittedly, this study was conducted in a clinic specializing in the assessment and treatment of neurodevelopmental disorders, which may have guided referral patterns and increased the likelihood of detecting ASD thereby inflating the comorbidity rates. Nevertheless, the findings provide support for the idea that SM and ASD are two disorders that can and frequently do co-occur.

A recent investigation by Klein et al. (2019) resulted in a similar conclusion. For 42 children 2 to 13 years of age who were referred to a university community clinic for individuals with communication disorders and who met the DSM-5 criteria for SM, parents and teachers completed the Behavior Assessment System for Children (BASC; Reynolds and Kamphaus 2015). The BASC is a standardized instrument for measuring internalizing problems, externalizing problems, and adaptive skills in young people. It also contains a clinical index comprising specific items referring to developmental social problems, atypicality, and withdrawal that are indicative of the possible presence of ASD (Volker et al. 2010; Zhou et al. 2020). The results showed that the children with SM displayed more internalizing symptoms and more problems with adaptive skills than externalizing symptoms. Most interestingly, however, the study revealed that 80% of the children scored above the cut-off on the autism probability index, with many of them displaying communication problems, persistent withdrawal, difficulties with developing and maintaining social relationships, and unusual behaviors. This finding led the researchers to conclude that further screening for ASD in children with SM is indicated.

In another study, Stein et al. (2011) examined whether SM and ASD share pathophysiological features. Participants were 99 nuclear families that included 106 children with SM for which a number of single nucleotide polymorphisms (SNPs) in the contactin-associated protein-like 2 (*CNTNAP2*), which is considered to be a susceptibility gene for ASD, were genotyped. The results indicated that the SNP *rs2710102* was significantly associated with the presence of SM. Interestingly, in a separate sample of young adults, the pertinent polymorphism also appeared to be accompanied by an increased risk of scoring high on social anxiety-related traits. On the basis of these findings, it was concluded that on a genetic level, SM, SAD, and ASD appear to share a similar susceptibility factor. Therefore, Stein et al. (2011) raised a cautionary flag regarding the classification of SM as a pure anxiety disorder by noting that there might be “considerable heterogeneity in the SM syndrome such that some forms … are more closely allied with the ASD spectrum and its association with *CNTNAP2*” (p. 830).

A recent investigation by Muris (2020) examined the relationship between SM and ASD in a non-clinical population. Parents of 172 children 3 to 6 years of age who were recruited via day care and preschool facilities as well as online platforms (e.g., facebook) completed a survey containing scales for measuring symptoms of SM, ASD, and SAD. The results showed that there were statistically significant positive correlations between SM and ASD (*r* = 0.43) as well as SAD (*r* = 0.67). These findings indicated that higher levels of SM symptoms were associated with higher symptom levels of both ASD and SAD. Most interestingly, hierarchical regression analysis revealed that ASD symptoms accounted for an extra proportion of the variance in SM symptoms (2%) after controlling for the influence of SAD symptoms, which emerged as the most robust predictor. These findings point out that although social anxiety appears to be the most prominent feature of SM, autism spectrum symptoms also make a significant contribution to this disorder.

There is also tentative support for the presence of ASD-related cognitive deficits in children with SM. Nowakowski et al. (2011) aimed to examine the quality of interactions between children with SM and their parents by focusing on joint attention processes. Joint attention can be defined as the shared focus of two individuals (in this case: the child and the parent) on an object or event that is achieved when one individual alerts another by means of eye-gazing, pointing, or verbal or non-verbal indications (Moore and Dunham 1995), which is an ability that plays a role in children’s acquisition of adaptive social behavior and has been found to be impaired in young people with ASD (e.g., Mundy 2016). In their study, Nowakowski et al. compared 19 children with SM, 18 children with mixed anxiety, and 26 children with a typical development (all in the age range of 5 to 8 years) with regard to their level of joint attention. Joint attention was assessed by direct coding of interactions between children and their parents who were observed in two experimental conditions (i.e., unstructured free play versus structured tasks such as talking about the child’s last birthday and preparing the child for a speech in front of a camera). No differences were noted among the three groups of children with regard to joint attention behaviors in the unstructured free play condition. However, under more structured conditions, children with SM and their parents were found to establish significantly fewer joint attention episodes following parental initiation as compared to children and parents in the mixed anxiety and the typically developing groups. Nowakowski et al. (2011) interpreted this finding in terms of stress-related coping behavior, suggesting that “children with SM may withdraw from their parents during stressful situations” (p. 78). However, it is also possible that children with SM exhibit a cognitive deficit that is similar to that observed in children with ASD, which of course would further underscore the relation between these two disorders.

Altogether, emerging evidence suggests that there is a relationship between SM and ASD. This is most clearly underscored by the study of Steffenburg et al. (2018) in which after a thorough clinical assessment a comorbidity rate between these two disorders as high as 62.9% was documented. In another sample of clinically referred children with SM, Klein et al. (2019) noted that no less than 80% surpassed the cut-off score of the autism probability index of the BASC-3. It should be noted, however, that the Steffenburg et al. study was conducted in a specific clinical setting in which diagnoses were obtained retrospectively. Further, the Klein et al. investigation relied upon a standardized questionnaire instead of a formal diagnostic instrument. Thus, both studies have their limitations. In other investigations much lower comorbidity rates have been reported (i.e., Kristensen 2000; 7.4%; Anderson and Thomsen 1998: 8.1%), but the methodology of these studies can be criticized because children with more severe forms of ASD were excluded.

Further research has shown that children with SM and children with ASD display overlap in social interaction impairments (Cholemkery et al. 2014) and appear to share a similar genetic liability (Stein et al. 2011). In addition, in typically developing children, a significant association was found between symptoms of both disorders even when controlling for concurrent symptoms of SAD (Muris 2020). Finally, tentative support has been obtained demonstrating that children with SM exhibit a specific cognitive deficit (i.e., impairments in joint attention) that is also characteristic for young people with ASD (Nowakowski et al. 2011). Obviously, the current body of evidence on the link between SM and ASD is still rather meagre, but it seems worthwhile to go beyond the artificial boundary that has been placed between both disorders and to conduct more research to investigate their relation in more detail.

## Commonalities and Differences in Social Difficulties of SM, SAD, and ASD

Apart from the observed links between SM and SAD/ASD, there is also evidence that children with ASD frequently display SAD (Davis et al. 2014; Spain et al. 2018; White et al. 2009a, b). Thus, SM, SAD, and ASD can be viewed as three allied psychiatric conditions. At a categorical level the three disorders frequently co-occur, while at a dimensional level symptoms of these disorders are substantially correlated and sometimes similarities are so prominent that it is difficult to distinguish them from one another (e.g., Kerns and Kendall 2012). In this section, we zoom in on various social difficulties associated with SM, SAD, and ASD with a focus on communalities as well as differences. More specifically, we will consider four aspects that are highly relevant for understanding social functioning and dysfunctioning: (1) emotional responses in social situations, or more briefly, social emotion, (2) social cognition, (3) social skills, and (4) social motivation (Pallathra et al. 2018).

### Social Emotion

Social emotion refers to emotional reactions that occur during social interactions, when being observed, or when performing in front of others. The reactions critically depend on thoughts, feelings, and actions of other people, which can either be experienced, recalled, anticipated, or imagined (Smith et al. 2006). With regard to our trinity of social disorders, most research is concerned with the basic emotion of anxiety. Obviously, this emotion reflects the key symptom of SAD (i.e., fear of negative evaluation and possible scrutiny by others) but also appears to be present in SM and ASD. It is important to note that anxiety as an emotion can be expressed in three response systems: namely subjective/cognitive, physiological, and behavioral (Lang 1968). When looking at anxiety in children with SAD, the prototypical picture involves a clear activation of all three response systems. In that case, children report high levels of subjective fear (i.e., “I feel really anxious”) and fear-related cognitions that are concerned with being negatively evaluated or scrutinized by others (e.g., “Others think that I am stupid”, “Others don’t like me”), report intense somatic symptoms such as palpitations, sweating, trembling, and blushing, and finally, withdraw from or totally avoid certain social situations (e.g., Stein and Stein 2008).

In children with SM, the picture is less clear. The earlier described study of Vogel et al. (2019) gives some clue about the subjective/cognitive experience of anxiety in children with SM: their fears and fear cognitions strongly resembled those of children with SAD, although some fear phenomena had an atypical, more idiosyncratic content (e.g., “I think my voice sounds funny”, “I don’t know how the conversation will evolve”). However, virtually nothing is known about the physiological and behavioral expression of anxiety in SM. It has been suggested though that, when facing a social situation that requires them to speak, children with SM physically become so tense that they resort to muteness as an avoidant strategy to reduce this physiological arousal. In a study by Young et al. (2012), some evidence was obtained for such a scenario. Five- to 12-year-old children with SM (*n* = 10, eight of whom were also diagnosed with SAD), SAD (*n* = 11, but none of whom had SM), or no psychiatric disorder (*n* = 14) were prompted (a) to have a conversation with an unfamiliar peer, and (b) to read aloud a story in front of a small audience consisting of an adult and a peer. It was found that in spite of the fact that children with SM (similar to those with SAD) exhibited significantly higher subjective fear levels in response to these socially challenging situations (in comparison to children with no diagnosis), they showed the lowest levels of physiological arousal. Young et al. (2012) tentatively concluded that “the decreased arousal displayed by these children represents successful avoidance of a distressful situation” (p. 539). This suggests that muteness, being the key feature of children with SM, can better be viewed on a symptom level rather than considered as a full diagnostic entity, which is an issue to which we will return later in this review.

As noted earlier, the expression of social anxiety in children with ASD is in part similar to but may also deviate somewhat from what has been noted in children with SAD without autistic characteristics. More precisely, on the subjective/cognitive level, many children with ASD indicate that they fear being scrutinized or negatively evaluated by others. On the physiological level, they experience high levels of arousal, and on the behavioral level, a clear tendency towards avoidance and withdrawal can be noted (Kerns and Kendall 2012, 2014). However, as noted by Ollendick and White (2012) there might be unique processes underlying the social anxiety of children with ASD, which may lead to a quite different emotional expression. In particular, the RRBIs exhibited by children with ASD could play a role here. For example, insistence on sameness (which can easily be elicited by unexpected changes in social events), difficulties with perceiving emotions of oneself and others (which can result in misinterpretation of social situations), and hypersensitivity to sensory input (which is particularly problematic in situations where a lot of people are present) are typical ASD symptoms that might fuel feelings of fear and anxiety and lead to frantic efforts to escape from or avoid certain social settings and increased self-injurious and aggressive behavior (Kerns et al. 2014). Although direct evidence for such scenarios is lacking, there is at least some research showing that high levels of social anxiety in children with ASD are associated with aggressive behavior (Pugliese et al. 2013).

Taken together, in terms of social emotion, similarities exist between SM, SAD, and ASD in that anxiety seems to be a prominent feature of the social functioning in children with each of these disorders. Meanwhile, there might be differences in the precise expression of anxiety: children with SAD display the prototypical picture of social anxiety (which is concerned with fear of negative evaluation and possible scrutiny by others), whereas in children with SM and ASD this emotion may also manifest itself in a more atypical way.

### Social Cognition

Social cognition can be defined as the capacity to perceive, interpret, and respond to the intentions, emotions, and behavior of other people and as such is concerned with cognitive processes that play a role in social interactions (Frith 2008). In the literature, four interrelated domains of social cognition have been identified, namely mentalizing (i.e., attributing mental states such as emotions, beliefs, and desires to other people), emotion recognition (i.e., inferring the emotional state of another person on the basis of facial, postural, or vocal expressions), social knowledge (i.e., awareness of social rules and norms in different social settings), and attributional style (i.e., the way people explain the course of social events; Pinkham et al. 2014). Within our trinity of social disorders, deficits in social cognition are most prominent in children with ASD. More precisely, children with autism spectrum problems appear to display clear deficiencies in all four domains of social cognition.

The most compelling evidence for this conclusion has been shown for the domain of mentalizing. Most of this research has focused on the concept of ‘theory of mind’, the meta-representational ability to impute mental states to oneself and others (Premack and Woodruff 1978). In their seminal study, Baron-Cohen et al. (1985) examined children with ASD (*n* = 20), children with Down’s syndrome (*n* = 14, who were comparable with the ASD children in terms of chronological and mental age), and normally developing children (*n* = 27) with an ingenious experimental paradigm named the Sally and Anne test. Briefly, during this test, children are presented with two doll protagonists, Sally and Anne. Sally has a marble, which she places in her basket. After she has left the scene, Anne secretly takes the marble and puts it in her own basket. Then Sally returns and the experimenter asks the critical question: “Where will Sally look for her marble?” Children who point at the previous location, appreciate that Sally has a ‘false’ belief about the situation and thus are able to employ a ‘theory of mind’. In the Baron-Cohen et al. (1985) study, it was found that whereas respectively 85% and 86% of the normally-developing and Down’s syndrome children successfully passed the test, only 20% of the children with ASD did so, which indicates that the vast majority of them did not show evidence of a ‘theory of mind’. The researchers concluded that children with ASD display a specific cognitive deficit that may account for the prototypical social impairments associated with this type of psychopathology. Further studies relying on other methodology have replicated that children with ASD exhibit clear deficits in the mentalizing aspect of ‘theory of mind’ (e.g., White et al. 2009a, b).

With regard to emotion recognition, there is also clear evidence that children with ASD have difficulties to infer emotions from other people’s facial, postural, or vocal expressions. Most studies have been conducted on the recognition of facial emotions. Although the results of this research are not univocal, a meta-analysis by Uljarevic and Hamilton (2013) indicated that individuals with ASD (this meta-analysis included child as well as adult populations) performed less well than control groups without ASD in correctly identifying the six standard basic emotions of happiness, anger, sadness, fear, disgust, and surprise. Although the effect sizes documented in this meta-analysis were quite heterogeneous across studies, the results indicated that there was no moderation effect of age, which confirms the notion that young people with ASD—just like their adult counterparts—have difficulties with recognizing the emotional expressions of other people (e.g., Fridenson-Hayo et al. 2016).

Few studies have been conducted with respect to the social knowledge of children with ASD. However, there is anecdotical evidence suggesting that very basic social rules such as “People act differently in public than they do in private” and “Know when you’re turning people off” are not obvious for persons with ASD (Grandin and Barron 2005). Further, an interesting study was conducted by Shulman et al. (2012) who presented children with ASD aged between 8 and 17 years (*n* = 18) and age- and IQ-matched typically developing children (*n* = 18) with a set of pictures depicting transgressions at school, of which some were socially inappropriate (e.g., sitting and eating on the floor) and others were morally condemnable (e.g., stealing from another student’s backpack). All children were asked to judge the appropriateness of various behaviors and to provide an explanation for their judgments. It was found that both groups of children were able to accurately describe and identify the unacceptable actions shown in the pictures. Typically developing children provided significantly more abstract rules for their judgments, whereas children with ASD more often gave non-specific condemnations (e.g., “You can’t do that!”) or an answer from an authority perspective (e.g., “The teacher will be really angry”). Further, the typically developing children were better in providing examples of situations in which the depicted behaviors would be acceptable, indicating that they were more flexible in applying social and moral rules than the children with ASD. Thus, although children with ASD appear to have some basic knowledge about the appropriateness of social behaviors, they tend to rely more on a fixed set of concrete rules, which will make them less sensitive to respond adequately to subtle signals defining the uniqueness of each social situation.

The final domain of social cognition that also appears to be impaired in ASD is concerned with children’s attributional style. Attributional style refers to the way that people interpret the course of a social event. To investigate this phenomenon in ASD, Klin (2000) employed the Social Attribution Task, an experimental paradigm that is based on a silent cartoon animation in which geometric shapes (i.e., a big triangle, a small triangle, and a small circle) enact a social play. Adolescents and adults with ASD (*n* = 40, including 20 participants with autistic disorder and 20 participants with Asperger’s syndrome) and normally developing adolescents and adults (*n* = 20) were shown the animation and asked to provide narratives about the social meaning of what was happening in the cartoon. The results indicated that the ASD group showed marked deficits across all aspects of social attribution as compared to the normally developing control group. More specifically, individuals with ASD identified less social elements in the cartoon animation, very infrequently made theory of mind-related attributions, more often included elements in their narratives that were irrelevant to the social plot, and were less able to ascribe personality features to the geometric shapes. The noted impairments in children with ASD were not related to verbal intelligence or level of linguistic skills and seemed to reflect “a clear sense of the impoverished social attribution abilities in this clinical sample” (p. 840).

In contrast to the wealth of empirical data on social cognition in ASD, research addressing mentalizing, emotion recognition, social knowledge, and attributional style in children with SAD is more sparse, while investigation of this topic is non-existent for children with SM. For children with SAD, a few studies have found evidence for the presence of deficits and impairments in theory of mind abilities (Banerjee and Henderson 2001; Colonnesi et al. 2017). For example, Banerjee and Henderson (2001) conducted an investigation in 63 8- to 11-year-old primary school children for whom they assessed levels of social anxiety and various social-cognitive abilities, including a standard false belief test (Baron-Cohen et al. 1985) and two—from a cognitive point-of-view—more complex tasks in which children were asked (a) to interpret a situation in which one person unintentionally commits a ‘faux pas’ (i.e., an embarrassing or tactless act or remark in a social situation) which upsets another individual (Baron-Cohen et al. 1999), and (b) to provide an explanation for deceptive self-representational displays used by story characters (e.g., pretending that one is not upset after getting hurt in a game with older children; Banerjee and Yuill 1999). It was found that children’s social anxiety levels were not significantly correlated with performance on the false belief task, indicating that social anxiety was not associated directly with any basic cognitive deficit in understanding other people’s mental states. However, statistically significant negative correlations were found between children’s social anxiety levels and their performance on the ‘faux pas’ and ‘deceptive self-representation’ tasks, which points out that high socially anxious children did experience at least some difficulties in understanding the complicated links between emotions, intentions, and beliefs.

Meanwhile, there are also indications that there are children with SAD who do not have poor theory of mind skills but rather show advanced capacity to impute mental states to oneself and others. Evidence for this idea comes from a recent study by Nikolic et al. (2019) who investigated the relation between social anxiety and children’s theory of mind ability in more detail. One-hundred-and-five children aged 8 to 12 years were assessed for social anxiety and mindreading using the ‘Reading the Mind in the Eyes’ test (Baron-Cohen et al. 2001a, b), which measures the accuracy of detecting mental states from the eye region of human faces. The results showed that while the average linear relation between social anxiety and mindreading was indeed negative, a curvilinear relation provided an even better fit. A close inspection of this relation revealed that high social anxiety was not only associated with poor mindreading skills but also related to advanced mindreading capacity. On the one end, there appears to be a group of children who show clear deficits in recognizing other people’s mental states. As a result, they have poor comprehension of what is exactly happening during social interactions, leading to confusion and unpredictability, which is the main source for their social fears and concerns. On the other end, there are also children who dispose of an advanced capacity to understand other people’s states of mind, which essentially is a positive thing (as it promotes successful social interactions) but may also come at a cost: these children can be extremely sensitive to other people’s opinions about them and have great awareness of the fact that they are subject to others’ attention and evaluation. This could result in heightened self-consciousness and fear of negative evaluation, which fuel feelings of social anxiety.

Several studies have explored whether there are impairments in emotion recognition in children with SAD; however, the results have been mixed. In one of the first investigations by Simonian et al. (2001), 15 children aged between 9 and 15 years who had a diagnosis of SAD and 14 age-matched control children were asked to identify the emotional expression depicted in a series of pictures of human faces. The results showed that children with SAD made more errors in correctly identifying the facial expressions of happiness, sadness, and disgust than the children in the control group. In another study, Wong et al. (2012) compared facial emotion recognition abilities across 7- to 13-year-old children with SAD, high-functioning ASD, or a typical development. It was found that children with high-functioning ASD were less capable of correctly identifying mild (but not extreme) affective facial expressions than the typically developing children, but the children with SAD scored in between and were not statistically different from the other two groups. There were clear methodological differences between the two studies, such as the use of different picture sets and variations in the intensity and presentation times of the facial expressions, meaning that the experimental boundaries for emotion recognition deficits in young people with SAD require further investigation.

No research can be found examining impairments in social knowledge of children with SAD and the same is true for deficits in the general attributional style of young people with this type of psychopathology. However, there is a substantial amount of studies showing that children with SAD or elevated symptoms of social anxiety display interpretation biases, which refers to the tendency to make a range of rather specific negative interpretations regarding themselves and other people when facing social situations. For example, Muris et al. (2000) examined such dysfunctional thinking in a sample of 252 primary school children of whom 28 were classified with SAD using a structured clinical interview. All children were exposed to a series of open-ended, ambiguous stories of social situations and for each of the stories they were instructed (a) to find out as quickly as possible whether that story reflected threat, (b) to tell how the story would end, and (c) to judge how they would feel when they would actually be confronted with that situation. The results showed that socially anxious children displayed lower thresholds for threat perception than control children, which means that they needed to hear fewer sentences of a story before deciding that it reflected threatening content. Further, socially anxious children more often thought that the stories would have a negative ending and also reported that they would have higher levels of negative feelings and thoughts in such situations, which points out that they had a tendency to interpret the ambiguous vignettes in a threatening way. A recent meta-analysis of all the research on the negative interpretation of ambiguity in young people with anxiety problems (Stuijfzand et al. 2018) indicated that the effect size of this so-called interpretation bias was largest for SAD as compared to all other anxiety disorders. Finally, there is recent evidence that children with ASD who also exhibit high fear and anxiety levels in social situations display similar threat-related interpretation biases, which suggests that social anxiety is the driving force behind this type of cognitive distortion (Neil et al. 2019).

In conclusion, there is convincing evidence that children with ASD show marked deficits in all domains of social cognition: compared to typically developing children, they perform more poorly on tasks requiring them to attribute mental states such as emotions, beliefs, and desires to other people (mentalizing) or recognize the emotional states of other persons (emotion recognition), tend to be less aware of rules and norms applying to various social settings (social knowledge), and have difficulties with correctly explaining the course of social events (attributional style). In children with SAD, the impairments in social cognition are in general less marked as compared to those noted for children with ASD; only in the domain of attributional style, children with SAD have been shown to clearly display a negative bias in the interpretation of (ambiguous) social situations. There is virtually no research with regard to the social cognition abilities of children with SM, which hence remains an important area of future inquiry.

### Social Skills

Social skills refer to a person’s competences facilitating the interaction and communication with other people, both verbally and non-verbally through gestures, body language, and personal appearance (Little et al. 2017). Given the diagnostic criteria for ASD, it will come as no surprise that children with this disorder tend to display poor social skills. Evidence for this notion comes from questionnaire-based studies in which parents and teachers evaluate the social abilities of children with ASD. This research has generally shown that children with ASD display lower levels of appropriate skills (e.g., smiling at other people, helping a person who is hurt, doing nice things for others) and higher levels of inappropriate skills (e.g., getting upset when having to wait, hurting other people’s feelings, interrupting others while speaking) as compared to typically developing children (Beighley and Matson 2014). Observational studies have also shown specific deficits in the social interaction and communication skills of children with ASD. For example, Macintosh and Dissanayake (2006) used a time sampling method to register and code the social behaviors of 39 high-functioning children with ASD aged 4 to 10 years and 17 age-matched typically developing children who were playing in the schoolyard. Although it was noted that children with ASD were capable of engaging socially with other children, the observations demonstrated that their social behavior deviated significantly from that of the typically developing children. For example, children with ASD were more often solitary, participated less in social play, communicated less with other children, and engaged less in enduring, reciprocal interactions with others (see also Murdock et al. 2007).

The social skills deficits of children with ASD may have serious consequences for the development of relationships with peers. An exemplary study on this topic was conducted by Kasari et al. (2011) who explored self-, peer-, and teacher-reports of social relationships in 60 high-functioning children with ASD in the primary school age (6 to 11 years) and 60 typically developing control children by means of questionnaires and a social network analysis. The results showed that children with ASD had fewer reciprocal friendships: only a minority of these children (18%) nominated a child as best friend, which was then confirmed by that peer. In typically developing children, this percentage of reciprocal friendships was significantly higher (64%). Further, it was found that the friendship quality of children with ASD was also lower than that of typically developing children: they spent less time with friends and were less open to share feelings. Finally, the network analysis revealed that children with ASD were more often isolated (13% versus 0%) or in the periphery of their classroom (42% versus 10%) than the typically developing children who were more frequently well-connected to their classmates (37% versus 58%) or even popular and central figures in their class (8% versus 32%). There are indications that the lack of social connection of children with ASD is not a transient phenomenon as their relationships with peers and other people often remain poor in terms of quantity and quality during adolescence and adulthood as well (Orsmond et al. 2004).

Another negative correlate of poor social skills is that children with ASD are prone to become involved in bullying experiences. Although some children with ASD are bullies themselves (e.g., Van Roekel et al. 2010), most become the victims of this type of negative social behavior (Schroeder et al. 2014). Because they tend to behave in socially awkward ways, they often attract the attention of peers some of whom approach them in a negative way. Given the poorly developed coping skills, their limited social network and friendships, and the tendency to display strong emotional reactions (e.g., visible anger, anxiety, or sadness) of children with ASD, perpetrators are not stopped but rather encouraged to commit their condemnable acts of physical aggression, verbal aggression, social exclusion, and cyberbullying. Studies that examined the occurrence of being a victim of bullying in children with ASD found rates of up to 94%, depending on the definition (e.g., verbal teasing versus physical aggression), time frame (e.g., past week versus past year), and the informant reporting on the experiences (e.g., teacher versus child). Most importantly, however, research including a typically developing control group has revealed that children with ASD are significantly more likely a target of bullying (Wainscot et al. 2008).

Thus, it is clear that the social skills of children with ASD are poorly developed and associated with various negative sequelae, but what do social skill deficits look like in children with SM and SAD? Studies examining social skills deficits in children with SAD have yielded quite mixed findings (Levitan and Nardi 2009). For example, in the investigation by Spence et al. (1999) who compared 27 clinically diagnosed children with SAD aged 7 to 14 years and a matched nonclinical control group, various measures of social skills and competences were used including self-, parent-, and teacher-reported questionnaires as well as a number of behavioral assessments (e.g., role plays with another child, a reading aloud task, and a natural observation of children’s interaction with peers). The results showed that the children with SAD not only had lower social skills scores on various questionnaires as compared to the control children, but also actually demonstrated these social skills deficits during some of the behavioral tasks. That is, the children with SAD responded with fewer words during the role plays and initiated less interactions with their peers at school. On the reading task, however, they performed just as well as the nonclinical control children. In another study by Cartwright-Hatton et al. (2005), non-clinical 10/11-year-old children with and without high social anxiety (*n* = 20 in both groups) were prompted to have a three-minute conversation with an unfamiliar adult. Afterwards their videotaped social skills were rated by themselves and by independent observers. It was found that the independent observers were unable to distinguish between the high and low socially anxious children. However, the high socially anxious children rated themselves as appearing less skilled than their low socially anxious counterparts. These results led Cartwright-Hatton et al. (2005) to conclude that children with SAD do not necessarily display social skills deficits, but rather tend to believe they perform less well in social situations, signifying the presence of a cognitive distortion. It is possible that the inconsistent results are due to the fact that SAD is not a homogeneous psychiatric condition, but rather consists of various subtypes (i.e., a subtype with clear social skills deficits and a subtype which has acquired adequate social skills but is dominated by negative cognition). Meanwhile, it should also be noted that the research conducted so far has relied on different methods to assess social skills deficits in specific circumstances. In their review of this topic (including research of both child and adult samples), Levitan and Nardi (2009) are in favor of the latter explanation when they conclude that “In general, the results indicate that socially anxious people perform more poorly in spontaneous social interactions than control participants, are classified by observers as less assertive, less friendly, and shy, but present only discrete differences in structured situations” (p. 702).

Only one study explored the social skills of children with SM (Cunningham et al. 2006). In that study, 58 children with SM on average 7 years of age and 52 community control children without psychiatric problems were compared on a number of socio-emotional variables, among which parent- and teacher-ratings of social skills and children’s self-reported social competence. Although children with SM perceived themselves as equally competent in social situations as control children, parents and teachers rated these children as less socially skilled and this was not only the case in situations that required speaking but also in situations in which they did not have to speak. Parents, for example, evaluated the children with SM as less confident in social situations, having more difficulties to make friends, and less likely to join groups. On the basis of these findings, Cunningham et al. (2006) concluded that SM is associated with comparable social skills deficits as are observed in some children with SAD.

Do the social skills deficits in children with SM and SAD also have repercussions for their daily social functioning? Most research has again been conducted on children with SAD and in general the results have shown that children with this anxiety disorder have more problems in establishing friendships, are in general less popular, and are also more frequently a target of victimization by their peers. For example, in a study by Scharfstein et al. (2011a, b), the interpersonal functioning among children with SAD, children with generalized anxiety disorder, and non-anxious control children (aged 6 to 13 years) was compared. It was found that children with SAD clearly had the lowest number of friends and experienced more difficulty in making friends than children with generalized anxiety disorder and non-anxious control children. In a further investigation by Baker and Hudson (2015), 39 children with SAD, 28 children with other anxiety disorders, and 29 nonclinical children first identified their closest friend and described some general features of this friendship (i.e., duration, frequency of contact). Following this, the children and their best friends evaluated the friendship quality by means of a standardized questionnaire. The results showed that while friendships did not differ in terms of general features across the three groups, children with SAD had significantly lower friendship quality as reported by themselves and by their best friend as compared to the children with other anxiety disorders. In another study, Gazelle et al. (2010) examined the symptom and diagnostic profiles of solitary children who had been identified by their peers in school (*n* = 192, with an average age of 8 years). Compared to an age- and demographically-matched control group, the solitary children were more often diagnosed with SM and SAD than children in the control group, which suggests that children with both diagnoses at a relatively young age run greater risk to become socially isolated.

With regard to peer victimization, there is clear evidence for a relation between SAD and falling victim to other children’s verbally and physically aggressive and socially exclusive acts. While the majority of the research has demonstrated that peer victimization experiences increase the risk for developing SAD and thus appear to play a causal role in this type of psychopathology (Pontillo et al. 2019), there is also support for a reverse scenario in which SAD acts as an antecedent of being bullied by peers (e.g., Hodges and Perry 1999; Pickard et al. 2018). In his well-known monograph on childhood bullying, Olweus (1993) also noted that the presence of SAD-related characteristics such as shyness and anxiety and the concomitant lack of social skills make children prone to become victim of bullying. For children with SM, only one study explicitly addressed this topic (Cunningham et al. 2004) and the results showed that these children had more problems in building friendships with peers but were not more frequently victimized by other children as compared to the control group. It should be noted, however, that the children in this study were still quite young (with an average age of 7 years) and so we don’t know to what extent bullying and victimization become more prominent in young people with SM during later development.

Altogether, children with ASD appear to display clear deficits in their social skills, which hinder them to successfully engage in social interactions, and may have serious consequences for establishing long-lasting friendships and also make them a target for peer victimization. There is evidence suggesting that the poor social skills of children with ASD are (at least partially) grounded in the social cognition deficits that have been described in the previous section. For example, in their cross-sectional study of 108 individuals with high-functioning ASD aged between 9 and 27 year, Bishop-Fitzpatrick et al. (2017) showed that after controlling for age and intelligence level, better social cognition abilities were significantly associated with higher levels of socially adaptive behavior and lower levels of social problems. Further, children with SM and SAD also exhibit shortcomings in their social skills, although one might expect that these deficits are more modest than in children with ASD. However, studies directly comparing the social skills of ASD and SAD/SM children are rare. One exception is the investigation by Scharfstein et al. (2011a, b) who conducted a detailed analysis of social behavior during structured role play interactions in 30 children with Asperger’s disorder, 30 children with SAD, and 30 typically developing children, all aged between 7 and 13 years. It was found that children with Asperger’s disorder performed equally well as typically developing children, and that only children with SAD exhibited significantly lower levels of social skills. However, an analysis of the vocal characteristics revealed that children with Asperger’s disorder deviated from typically developing children and children with SAD because they displayed a distinct pattern of speech: that is, they spoke more softly and had a lower vocal pitch and less vocal pitch variability, which can be subjectively heard as monotonic talking. The study by Scharfstein et al. (2011a, b) seems to warrant the conclusion that although there are only subtle differences in the socially interactive behaviors of children with ASD and SAD, as the two groups did not dramatically differ in terms of social skills. However, it should be noted that this study included ASD children with a fairly high level of intelligence (mean IQ = 114), and there are indications that cognitively high-functioning children with ASD are capable of compensation and display good skills in social situations (Livingston et al. 2019). Moreover, in the Scharfstein et al. (2011a, b) study, social skills were assessed during a series of structured role plays, and so it remains to be seen whether similar results would be obtained for children with ASD who face real-life social situations. Thus, more research comparing the social skills (deficits) between children with ASD and children with SAD and in particular children with SM is certainly needed.

### Social Motivation

Social cognition and social skills to a large extent determine to what extent children are capable of engaging in interactions with other people. Social motivation is another aspect of social functioning that refers to the need and willingness to interact with others and to be accepted by them. It is generally assumed that human beings in general have a natural need to belong with others and to relate with them (Baumeister and Leary 1995), but there are also clear individual differences with regard to this need that may be mediated by certain types of psychopathology. SAD is thought to be not really associated with deficits in social motivation, which can be derived from the fact that socially anxious people seek treatment because they feel unhappy about their relationships with other people. Although direct evidence for the social motivation tendencies of individuals with SAD is sparse, there is an interesting recent study by Goodman et al. (2019) who assessed the personal strivings in 41 adult individuals with this anxiety disorder and 43 healthy controls. Among the list of personal strivings types, four were of a social nature, namely ‘affiliation’ (i.e., concern for or desire to establish, maintain, or repair friendships), ‘interpersonal’ (i.e., an objective or goal focused on others), ‘intimacy’ (i.e., commitment and concern for others, quality of relationships rather than quantity), and ‘self-presentation’ (i.e., making a favorable impression on others). Although the individuals with SAD reported greater difficulty in pursuing their strivings, the content and frequency of these personal goals were similar to those noted for the healthy controls. Of course, it needs to be explored whether these findings also apply to children and adolescents, but at least they suggest that the social motivation of adults with SAD is intact.

The latter is also supported by the fact that SAD is accompanied by a number of clinical correlates that strongly suggest there is a discrepancy between the social motivation and the social accomplishments of a child. Feelings of loneliness are a case in point. This unpleasant feeling that occurs as a result of a lack of connection and communication with other individuals is quite prevalent in young people with SAD. For instance, Maes et al. (2019) conducted a meta-analysis on the relationship between social anxiety and loneliness in childhood, using the data of 102 cross-sectional studies and 10 longitudinal investigations. The results indicated that there was a substantial positive association between social anxiety symptoms and feelings of loneliness (average effect size: *r* = 0.46), and this link did not vary in strength between children and adolescents. Further, the analysis of the longitudinal studies included in the meta-analysis revealed that there were reciprocal associations between social anxiety and loneliness over time, indicating that social anxiety predicted subsequent feelings of loneliness and that feelings of loneliness in turn predicted subsequent levels of social anxiety. In a similar vein, it has been shown that children with SAD are also more prone to develop depression. An example is the well-cited study by Stein et al. (2001) who prospectively investigated the prevalence of SAD and depression in a sample of 2548 adolescents and young adults aged 14 to 24 years. It was found that SAD at baseline was associated with an increased likelihood (odds ratio = 3.5) of depressive disorder onset during the follow-up period of 3 to 4 years. Thus, research has demonstrated that SAD in young people is associated with higher levels of loneliness and depression (see also Danneel et al. 2019).

For SM, only indirect, anecdotal information can be found regarding social motivation. For example, Walker and Tobbell (2015) conducted detailed online interviews with four adults who had been diagnosed with SM during their childhood years. The general themes in the accounts of their subjective experiences with the disorder reflected “loneliness” and “loss”. That is, the adults indicated that the SM had isolated them from other people and this had seriously hindered them in academia, work, and personal life. The dissatisfaction and negative affect associated with these experiences indicate that they had wanted a different social life and suggests that they were socially motivated (see also Omdal 2007). Further, there is some evidence showing that depression is a frequent comorbid disorder in young people with SM. Illustrative is a study by Gensthaler et al. (2016b) who compared the comorbidity patterns in young participants with SM (*n* = 95) or SAD (*n* = 74), all aged between 3 and 18 years. It was found that major depression was present in 12% of the participants with SM (as compared to 26% in those with SAD), and that this diagnosis was always made during the adolescent years (i.e., between 12 and 18 years). Of course, depression can arise from a wide range of etiological factors, but there are clear indications that in young people social isolation and peer problems are important determinants of this disorder (Hammen 2009).

Because children with ASD engage in less eye contact, are less sensitive to the feelings and thoughts of others, and often withdraw from social situations, one might conclude that they have “a powerful desire for aloneness” as suggested early on by Kanner (1943, p. 249). The apparent lack of social interest has prompted some scholars to argue that ASD can best be regarded as “an extreme case of diminished social motivation” (Chevallier et al. 2012, p. 231). Experimental researchers have tried to underpin this point of view by demonstrating that individuals with ASD display deficits in the processing of social rewards. For example, Scott-Van Zeeland et al. (2010) used a reward learning task presented on the computer during which 16 high-functioning boys with ASD (average age = 12 years) and 16 typically developing boys had to classify abstract fractal-like images into two groups. Following the classification of each of the images, children received feedback on their performance in three ways. The neutral feedback merely consisted of the words “Correct” or “Incorrect” shown on the computer screen after children had given their response, the monetary feedback consisted of the presentation of a picture of three gold coins with the word “Correct” or a picture of three red X’s through the gold coins with the word “Incorrect”, while the social feedback consisted of a picture of a smiling woman with the words “That’s right” or a woman with a sad face with the words “That’s wrong”. Pictures and feedback combinations were presented to the children in two experimental runs of 72 trials each: one run involved the contrast of monetary reward versus neutral feedback, whereas the other run pertained to the contrast of social reward versus neutral feedback. During the learning task, scans were made of children’s brain activity using functional magnetic resonance imaging (fMRI) to investigate the neural circuitry underlying reward learning. The results showed that the typically developing children displayed a significant improvement in their classification accuracy over the course of the experiment, and this occurred independent of the way that the feedback (neutral versus social or monetary reward) had been given. Children with ASD continued to perform on a chance level throughout both runs of the experiment. This indicates that while typically developing children show clear signs of learning following feedback, this process appears to be impaired in children with ASD. Even more interestingly, when looking at the neural responses to rewards, it was found that children with ASD exhibited diminished neural responses in the ventral striatum (a part of the brain situated in the subcortical basal ganglia of the forebrain, known to play a prominent role in reward processing) to both monetary and social rewards, with a more pronounced reduction being noted in response to social rewards. This appears to indicate that children with ASD are less sensitive to feedback—in particular of a social nature, and could help explain why they are less interested to engage in relationships with other people, which is the key tenet of the social motivation theory (Chevallier et al. 2012).

Meanwhile, it should be noted that the social motivation theory has been seriously challenged for several reasons. To begin with, a recently conducted meta-analytic review (Clements et al. 2018) has revealed that the hypoactivation in certain brain areas is less specific than suggested by the results obtained in the Scott-Van Zeeland et al. (2010) study. More precisely, the atypical processing noted in ASD was not shown to be stronger in response to social rewards than to non-social rewards, which implies that evidence obtained by this type of research is not entirely supportive for motivation in the social domain but is in need for a more general explanation. Moreover, Jaswal and Akhtar (2019) recently argued that it is questionable to assume on the basis of behavioral characteristics and even aberrant brain processing patterns that children with ASD are less socially motivated. They referred to testimonies of children and adults with ASD from which it can be inferred that individuals with this type of psychopathology do want to connect and interact with other people, but do so in an unconventional or idiosyncratic way. This notion is supported by findings indicating that (a) children with ASD tend to report that they feel lonely, and that (b) a substantial proportion of them develop depression. With respect to the former, a study by Bauminger et al. (2003) investigated the level of social interaction with peers and feelings of loneliness in 18 high-functioning children with ASD (aged 8 to 17 years) and 17 age- and IQ-matched control children. Observations in natural settings revealed that the children with ASD spent only half of the time in social interactions with peers as compared to the control children. Further, children with ASD reported significantly higher levels of loneliness than the control children, and these feelings not only pertained to the low frequency of social involvement with others (i.e., social loneliness) but also to the negative affect associated with the lack of social contact (i.e., emotional loneliness). Finally, in both children with ASD and typically developing children, negative associations were found between social competence and loneliness, which indicates that success in social interactions decreases the likelihood of this negative affective reaction (see also Bauminger and Kasari 2000; Deckers et al. 2017; White and Roberson-Nay 2009). Concerning the occurrence of depression in ASD, it has been noted that 7.7% of the children with this neurodevelopmental disorder come to suffer from a depressive disorder before the age of 19 and this rate is about 4 times greater than that documented in the general population (Hudson et al. 2019). Similar to the developmental patterns found in non-ASD youth, the likelihood for developing depression in young people with ASD seems to increase in adolescence (Pezzimenti et al. 2019) and it is highly plausible that the prototypical social impairments and associated social problems are among the factors that underlie this increased risk (Magnuson and Constantino 2011).

In conclusion, young people with SM, SAD, and ASD all display behaviors that could be viewed in terms of a lack of social interest and motivation. However, in children with SM and SAD it is likely that these behaviors are fueled by fear and anxiety which result in persistent avoidance of (some) social situations. In children with SM who are often less communicative about their fears and anxieties, this may be less obvious leading to questions about their social motivation. In children with ASD, it is clear that deficits in information processing undermine their sensitivity and responsivity to social cues, and as a result these children are less likely to orient toward, seek out and enjoy, and attempt to maintain relations to other people (Chevallier et al. 2012). Meanwhile, Jaswal and Akhtar (2019) noted that despite this reduced quantity of social interest, children with ASD do engage in interactions with other people although they might do this in a different manner. If these attempts are not met in a satisfactory way, they may respond with negative affect, showing signs of loneliness and depression just like their typically developing peers. There is evidence that this is particularly true for high-functioning children with ASD who are to some extent capable of interacting with peers but due to their social peculiarities are less successful in initiating and maintaining social relationships (e.g., Pezzimenti et al. 2019).

To recap, there appear to be considerable impairments in the social functioning of children with SM, SAD, and ASD, although differences in the extent to which various domains are affected have also been noted. Our review has demonstrated that most research has focused on the social difficulties in children with SAD and ASD. Far less studies have been conducted on SM, but it plausible to assume that—given its relations to SAD and ASD—children with this psychiatric condition also display comparable problems in social functioning. Obviously, this is an important topic of future investigation as more knowledge of social emotion, social cognition, social skills, and social motivation would increase our understanding of SM and could—as we will see later—yield important leads for treatment.

## RRBIs and the Severity of Social Problems

As noted earlier, RRBIs are an integral, core component of ASD and these features are positively related to severity of the social problems in individuals suffering from this neurodevelopmental disorder. That is, a stronger presence of RRBIs is typically associated with greater inflexibility and rigidity that may have a direct negative impact on the social behavior and also hinder the emergence of adequate emotion regulation strategies in response to social situations (Lam et al. 2008).

A case in point is insistence on sameness, which is considered as an important domain of RRBIs (Leekam et al. 2011). Insistence on sameness refers to an overreliance on rigid, routinized, and ritualistic behaviors that likely leads to a reduced exposure to novel and/or challenging situations (Bishop et al. 2013). Note that the latter bears similarity to the avoidance behavior that is assumed to play a critical role in the maintenance of anxiety disorders such as SM and SAD. Indeed, there is evidence indicating that insistence on sameness is significantly and positively associated with global anxiety scores in young people with ASD. For example, in a recent study by Lidstone et al. (2014), parents rated their 3- to 17-year-old offspring with ASD using the Repetitive Behavior Questionnaire (Leekam et al. 2007), which measures two main components of RRBIs: insistence on sameness and repetitive behaviors, and an anxiety scale for measuring DSM-defined anxiety disorder symptoms in children (Spence 1998). The results showed that the insistence on sameness factor correlated significantly and positively with the total anxiety score, whereas the repetitive behaviors factor did not. Comparable findings have been obtained in other studies (Factor et al. 2016; Gotham et al. 2013; Russell et al. 2019; Uljarevic and Evans 2017; Uljarevic et al. 2017b). Although insistence on sameness has been shown to be positively associated with global anxiety, two other studies have found that this type of RRBIs is less convincingly linked to social anxiety in children with ASD (Black et al. 2017; Rodgers et al. 2012). This of course suggests that this construct is less relevant for understanding SM and SAD as comorbid conditions in this population. It should be borne in mind, however, that in these studies social anxiety was assessed by means of a parent-rating scale which may be less suitable for assessing the atypical/idiosyncratic manifestations of this anxiety problem in children with ASD. In addition, oftentimes, parents note that their offspring with ASD have social problems but do not label this as social anxiety as these children are typically less communicative about their symptoms.

There is also a considerable amount of research indicating that the relation between insistence on sameness and anxiety is mediated by the dispositional trait of intolerance of uncertainty (e.g., Glod et al. 2019; Hwang et al. 2020; Joyce et al. 2017; Wigham et al. 2015), which can be defined as the tendency to react negatively to situations and events that are unforeseen and unpredictable (Buhr and Dugas 2006). This concept has its origin in the anxiety literature and is strongly associated with clinically significant anxiety problems including SAD. It is suggested that individuals scoring high on intolerance of uncertainty have a tendency to interpret ambiguous information as threatening and as a result have a stronger inclination to avoid all kinds of situations (Carleton 2012). Interestingly, children with ASD score significantly higher on intolerance of uncertainty than typically developing children (Boulter et al. 2014; Vasa et al. 2018). This is one reason why this factor has been put forward as an important cognitive mechanism behind the anxiety symptoms and in its wake pathological demand avoidance (i.e., the obsessional avoidance of the demands of everyday life; Newson et al. 2003) of children with this neurodevelopmental disorder (Maisel et al. 2016; Stuart et al. 2020).

The insistence on sameness that can be noted in children with ASD may directly fuel avoidance as well as elicit other challenging behaviors such as edginess and irritability, as a result of which the social communication is further complicated (Bitsika and Sharpley 2016). But also on a cognitive level these children are often characterized by rigidity, accounting for the fact that they often display a greater proneness to worry (e.g., Gillott et al. 2001; White et al. 2014a, b) and rumination (e.g., Mazefsky et al. 2013, 2014). Burrows et al. (2017) have described a neuropsychological model in which three putative brain networks are put forward to explain why children with ASD are so susceptible to high levels of negative thinking: (1) a salience detection network, involving the dorsal anterior insula, the dorsal anterior cingulate cortex, and subcortical structures including the amygdala, which signals the (potential) presence of social threat and contributes to the coordination of the subsequent emotional and cognitive response, (2) an executive control network, consisting of nodes in the posterior parietal cortex and the dorsolateral prefrontal cortex, which serves the regulation of attentional processes, and (3) a default network, composed of cortical areas such as the medial prefrontal cortex, subgenual anterior cingulate cortex, and posterior cingulate cortex, that subserves internally directed self-referential thinking. The idea is that in ASD the salience detection network becomes (too) easily activated, which then due to attentional control deficits (executive control network) results in difficulties disengaging from threat, ultimately causing prolonged self-referential processing in the default mode network, the outcome of which is high levels of repetitive thinking. Thus, the cognitive inflexibility that is so frequently observed in children with ASD is hypothesized to be mediated by aberrant brain processes, and also provides a plausible explanation for why social problems (in this example: social emotion) might be more persistent in young people with this type of psychopathology (Burrows et al. 2017).

Sensory processing abnormalities are a special class of RRBIs that have less in common with the two main factors of repetitive behaviors and insistence on sameness (Uljarevic et al. 2017a). In fact, it has been suggested that the prototypical repetitive behaviors and resistance to change of children with ASD are often moderated by unusual sensory experiences (e.g., Baker et al. 2008; Joosten et al., 2009). Most pertinent to the present article is the observation that sensory processing abnormalities may also contribute to anxiety symptoms. For example, several studies have noted that in particular sensory hypersensitivity is positively associated with anxiety, stress, and other socially undesirable symptoms in young people with ASD (Mazurek et al. 2013; Neil et al. 2016; Uljarevic et al. 2016).

There is some evidence that sensory hypersensitivity is not only relevant for ASD but may also play a role in individuals with SM and SAD. Hofmann and Bitran (2007), for example, explored the relationship between sensory processing sensitivity, as measured by the Highly Sensitive Person Scale (Aron and Aron 1997), and anxiety and avoidance in 89 adult patients with SAD referred to an outpatient treatment center. The results showed that sensory hypersensitivity was not significantly correlated with social anxiety symptoms in general but did show a positive correlation with avoidance behavior, which may indicate that hypersensitivity is associated with greater interference in daily functioning (Hofmann and Bitran 2007). For SM, direct empirical evidence for a link with sensory hypersensitivity and symptom levels is lacking, but it has been reported that some children with this anxiety disorder do not speak because they report to experience that their “voice sounds funny” (Bar-Haim et al. 2004). It has been suggested that this may be partly due to aberrations in the auditory monitoring and regulation of self-vocalization (Muchnik et al. 2013), which might be a special case of highly sensitive sensory processing.

In conclusion, while the trinity of SM, SAD, and ASD have a lot in common with regard to the phenomenology of social difficulties and their underlying processes, children with ASD appear to display a special set of symptoms, the RRBIs, of which insistence on sameness and sensory hypersensitivity are particularly important as they are likely to promote social anxiety or elicit other challenging behaviors that hinder social interaction. It should be kept in mind that in the current classification system of the DSM, ASD is conceived as a spectrum disorder (APA 2013). This implies that its prototypical symptoms to a greater or lesser extent are present in all children, including those that do not fulfill the diagnostic criteria of ASD and thus may have a normal development. Indeed, there is empirical evidence showing that RRBIs do occur in typically developing children (although to a lesser degree than in children with ASD; Arnott et al. 2010; Barber et al. 2012; Joseph et al. 2013; Leekam et al. 2007). Thus, the adverse consequences of RRBIs such as insistence on sameness and sensory hypersensitivity are not only relevant for children with ASD but may also have a negative impact on the social functioning of typically developing children and children with other psychopathologies such as SM and SAD.

## SM: More than Just an Anxiety Disorder?

In current classification systems such as the DSM (APA 2013) and the ICD (WHO 2018), SM has been included in the category of anxiety disorders. This decision is justified since fear and anxiety appear to be primary features of the disorder. As we have noted in this review, many children with SM also have a comorbid anxiety disorder or, in the least, experience fear, anxiety, and fear-related cognitions in situations in which they are expected to speak. In particular, the relation with social anxiety appears to be quite robust: SAD is by far the most frequently diagnosed concurrent anxiety disorder in children with SM and the fear, anxiety, and fear-related cognitions of these youngsters are typically guided by social evaluative concerns. That is, they fear being exposed to possible scrutiny by others, just like children with SAD. Because of the intimate link between SM and SAD (Chavira et al. 2007), scholars have argued that SM might be a special variant of SAD (Bögels et al. 2010), with some of them viewing it as a developmental precursor of SAD (e.g., Bergman et al. 2002) and others considering it as an extreme manifestation of this anxiety disorder (e.g., Black and Uhde 1992). There seems to be support for both accounts. For example, the early age-of-onset noted for SM (APA 2013) in combination with the observation that full muteness usually disappears when children become older (Remschmidt et al. 2001) supports the developmental precursor hypothesis. Meanwhile, there is also evidence showing that children with SM display even higher levels of social anxiety symptoms than children with SAD (e.g., Young et al. 2012), which is in line with the extreme manifestation hypothesis. Thus, not speaking could reflect a rather primitive strategy displayed by young children in an attempt to deal with the fear and apprehension elicited by certain social situations, but could also represent the most extreme manifestation of anxious avoidance in situations in which one is clearly expected to speak but from which physical escape is impossible (e.g., school).

**Fig. 1:**
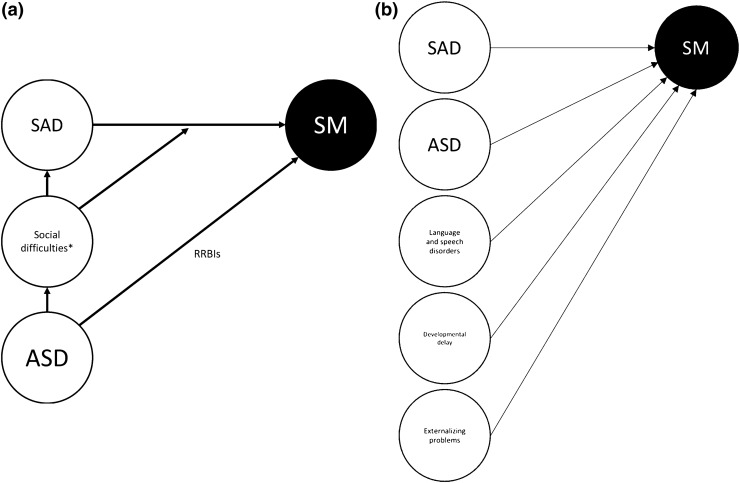
**a** Model displaying the potential role of ASD in the etiology of SM. As can be seen, both the social difficulties (partly via social anxiety) and RRBIs associated with this neurodevelopmental disorder may contribute to the persistent muteness displayed by children with SM. **b** SAD, ASD and a number of other (hypothesized) possible mental health and developmental conditions related to SM. *ASD* autism spectrum disorder, *SM* selective mutism, *SAD* social anxiety disorder, *RRBIs* Restrictive and Repetitive Behaviors and Interests; *Social difficulties encompass various domains: social emotion, social cognition, social skills, and social motivation

While the role of anxiety in SM is clear (e.g., Driessen et al. 2020; Muris and Ollendick 2015; Sharp et al. 2007; Viana et al. 2009), the purpose of the present review is to suggest that—in some children with SM—ASD seems to be present as well. We have pointed out that on a categorical and dimensional level—relations among SM, SAD, and ASD are substantial and they can be considered to be a unified trinity of social disorders. We have described shared social difficulties but also highlighted differences and nuances in the symptom picture of these three psychopathologies. We assume that relations among these disorders are not random but rather propose that SM should be conceptualized as a psychiatric condition that primarily emanates from SAD, but that in some cases ASD also makes a significant contribution. More precisely, it can be assumed that the pervasive social difficulties associated with this neurodevelopmental problem (e.g., social skills deficits) will fuel social anxiety symptoms as well as prompt muteness as the preferred strategy to deal with the excessive symptomatology elicited by specific social situations. Further, the RRBIs, especially the rigidity and cognitive inflexibility associated with insistence on sameness as well as sensory hypersensitivity, will enhance the social difficulties thereby further intensifying the social anxiety, but also promoting the persistent non-speaking behavior displayed by children with SM (see Fig. 1a).

Although the present review has focused on the role of social anxiety and autism spectrum problems in SM, it is important to note that in the psychological literature a number of other (psycho)pathological conditions have been identified that possibly contribute to the prototypical muteness associated with this disorder as well (see Fig. 1a). For instance, Steinhausen and Juzi (1996) noted that a considerable proportion of children with SM (38%) displayed premorbid speech and language disorders, and in particular articulation disorder (20%) and expressive language disorder (28%) were relatively common. In a similar vein, the study by Kristensen (2000) indicated that children with SM more often displayed developmental delay in the domains of motor (48.1%) and language (51.9%) as compared to typically developing control children (7.4% and 11.1%, respectively). It is easy to see how difficulties in speech and language as well as developmental impediments can cause children to become apprehensive of school and other social situations. Meanwhile, it is important to be aware of the fact that there is also research showing that not all children with SM are characterized by marked language and speech impairments and developmental delays and that—if present at all—deficits in these areas of functioning are rather subtle (e.g., Manassis et al. 2003). A similar point can be made regarding disruptive behavior problems. The earlier described study by Cohan et al. (2008) found that there is a subgroup of children with SM in whom heightened levels of externalizing problems are present as well. In particular, parents have sometimes described children with SM as stubborn, noncompliant, disobedient, oppositional, negative and manipulative, and it is easy to link each of these characteristics to the non-speaking behavior of their offspring (e.g., Diliberto and Kearney 2016). There is indeed research confirming that externalizing problems such as oppositional-defiant disorder are more prevalent among children with SM (e.g., as compared to children with SAD: 29% versus 5%; Yeganeh et al. 2006), but there are also studies showing that there are no signs of elevated levels of behavioral problems in young persons with SM (e.g., Cunningham et al. 2004,2006).

In previous publications, we (Muris and Ollendick 2015) and others (e.g., Cohan et al. 2006; Viana et al. 2009) have adopted the developmental psychopathology perspective to conceptualize the etiology of SM. One important premise of developmental psychopathology is that disorders like SM do not arise as a result of a single deterministic variable, but rather develop due to a set of risk and vulnerability factors that increase the probability of the psychiatric condition to occur. It is also important to bear in mind that the precise constellation of risk and vulnerability factors may be, and likely are different for each individual child, which is known as the principle of equifinality (Cicchetti and Cohen 1995). Thus, besides (social) anxiety other variables play a role in the development and maintenance of the non-speaking behavior of children with SM. This has not only been shown in the earlier described study by Cohan et al. (2008) but also by Mulligan (2012) who identified five subtypes of the disorder that were each typified by a specific set of underlying difficulties. Although anxiety appeared to be the prominent feature of one subtype of children with SM, other subtypes were characterized by the presence of other problems (e.g., developmental delays, oppositional behavior, language expression difficulties, sensory/self-regulation problems).

In addition, it is relevant to note that research has identified a number of other variables that are likely to be involved in the pathogenesis of SM. Strongly guided by the current notion that SM is an anxiety disorder—or even a variant of SAD, it is not surprising that the focus in past research has been mainly on variables that are thought to play a role in this type of psychopathology, including learning experiences (conditioning and avoidance), temperamental vulnerability (BI), anxiety-promoting rearing behaviors (overprotection and anxious rearing), genetic transmission of anxiety or anxiety-related traits, or other circumstances that cause the child to become apprehensive of speaking in challenging social situations (such as having to speak at school in a non-native language and bullying experiences; for a detailed overview of the literature, see Muris and Ollendick 2015).

However, we want to emphasize the point that SM is not always a pure anxiety phenomenon, and that—at least in a significant proportion of the children—other problems play a role and most importantly that ASD may also be present. Children with this neurodevelopmental disorder display a variety of social difficulties: besides intense emotional reactions to social situations and associated avoidance behavior, they also exhibit marked impairments in social cognition and social skills as well as a lack of social motivation. In the case of SM, this could mean that a child with ASD does not speak only because of fear and anxiety (or associated emotions such as shame and anger) but also because he/she does not properly understand specific social situations, has difficulties to read other people’s minds, does not know how to respond to the other person(s), and/or is less interested in engaging in the social interaction. Further, the RRBIs, as a specific set of ASD symptoms, could further hinder the process because the child is lacking the cognitive flexibility to manage the social situation or because the sensory hypersensitivity makes the social situation an even more aversive experience (Green and Ben-Sasson 2010).

The contribution of ASD to the etiology of SM does not mean that the anxiety pathway to the non-speaking behavior of children with this disorder is no longer or less relevant. It is plausible that there are clear commonalities in the processes underlying the social difficulties as noted for SM, SAD, and ASD (i.e., temperament, emotion regulation, and neurocircuitry), and even some of the specific difficulties in children with ASD may exert their influence on muteness via the anxiety route. For example, it can be assumed that the pronounced social skills and social cognition deficits of children with ASD will elicit expectations of negative evaluation and subsequent fear and anxiety, which in turn will lead to non-speaking behavior. However, there may also be specific issues in children with ASD that result in muteness without a mediating role of anxiety. Think in this regard of social motivational motives of non-speaking behavior, such as not being interested in the other person or not wanting to abort a routine or preferred activity, or a RRBI such as sensory hypersensitivity (e.g., the child does not to speak in class because it is already too noisy).

Altogether, in our view, ASD and—because this disorder is now conceptualized on a continuum—ASD-related traits likely play a role in the pathogenesis of SM. It is unclear why this point has been largely neglected in the psychological and psychiatric literature. Steffenburg et al. (2018), who recently conducted their systematic study on the comorbidity between SM and ASD, noted that because muteness is such a prominent and dramatic symptom, the diagnosis of SM alone is often made by clinicians as this classification directly and fully covers this key symptom (a phenomenon that is known as ‘diagnostic overshadowing’; Kanne 2013). Further, as mentioned before, in diagnostic systems such as the DSM and ICD, disorders have long been considered as distinct categorical entities, thereby excluding comorbidity as much as possible. This is still echoed in one of the diagnostic criteria of SM which states that the disturbance should not occur exclusively during the course of autism spectrum disorder (APA 2013). However, this criterion is rather difficult to maintain when acknowledging the more accepted notion that psychopathological phenomena (such as anxiety, mutism, and even ASD symptoms) are dimensional in nature (e.g., Hudziak et al. 2007; Krueger and Piasecki 2002). In relation to this point, we see no reason why ASD is listed as an exclusion criterion for one anxiety disorder—SM—while being allowed as a comorbid condition for other anxiety disorders including SAD. Finally, for a long time, researchers and clinicians have struggled with the objective classification of ASD. Although in the past decades reliable and valid diagnostic instruments such as the Autism Diagnostic Observation Schedule (ADOS; Lord et al. 1999) and the Autism Diagnostic Interview (ADI; Lord et al. 1994) have become available (Huerta and Lord 2012), their use in clinical settings is still no common practice (e.g., Brett et al. 2016). In addition, in particular when intelligence is higher and language skills are fairly good, the symptoms of ASD may be more masked, resulting in difficulties with making the proper diagnosis (Mazzone et al. 2012).

## Clinical and Research Implications

The acknowledgment of the involvement of ASD in the etiology of SM has a number of consequences for clinical practice with regard to the classification, assessment, and treatment of children with this disorder. In addition, this will have repercussions for future empirical studies on SM. In this section, these clinical and research issues will be discussed.

### Implications for Classification

With respect to the classification of SM in current diagnostic systems such as DSM (APA 2013) and ICD (WHO 2018), we think that its current categorization as an anxiety disorder is appropriate given the compelling empirical evidence on the role of (social) anxiety in this psychiatric condition. Future editions of these systems might consider the role of anxiety by including a specific criterion such as “avoidance of speaking due to anxiety, fear, or other distress elicited by specific social situations” to explicitly reflect this notion, thereby giving SM a more firm position within the family of anxiety disorders while also referring to its intimate relation with SAD in particular. Meanwhile, the potential contribution of ASD to SM also needs to be acknowledged in a more direct way. This could be most effectively done by (1) deleting the rather vague and ambiguous “the disturbance does not occur exclusively during the course of ASD” statement from the current criteria of SM, and (2) no longer considering SM as a differential diagnosis of ASD, as it is extremely difficult to establish the absence of social reciprocity and communication problems and RRBIs because these reflect a continuum. Instead, it would be preferable to simply allow SM and ASD to be comorbid conditions (just like is currently done with SM and SAD, and ASD and SAD). After all, classification systems are meant to help scientists and clinicians correctly identify the psychopathology of a child, to get a better understanding of the etiological mechanisms involved, and to arrive at a good indication for the proper treatment or intervention (Muris 2019).

The ongoing discussion on the defining criteria of SM illustrates some of the problems that we encounter in contemporary classification systems of mental disorders. More precisely, systems such as the DSM and ICD are originally based on the notion that psychopathologies are categorical in nature and that each disorder is defined by a unique set of symptoms that demarcate it from other psychiatric conditions. As noted earlier, mental disorders can better be viewed as continua with low symptom levels on the one end and high symptom levels on the other end (Hudziak et al. 2007; Krueger and Piasecki 2002). Moreover, relations across disorders and overlap in symptoms are the rule rather than the exception (e.g., Plana-Ripoll et al. 2019). Even a prototypical symptom such as mutism is not exclusively linked to the anxiety disorder SM but can be associated with a wide range of other mental health and developmental problems (see also Fig. 1b). As posited in the present review, we think that—among these—ASD also deserves its position and should not be excluded as a related condition. Meanwhile, it is certainly not our intention to make the assertion that SM is always explained by the presence of ASD. The role of (social) anxiety in SM has been demonstrated and hence should be the primary target in terms of assessment and treatment. However, in some children with SM, ASD may be implicated as well and thus also require clinical attention.

**Fig. 2:**
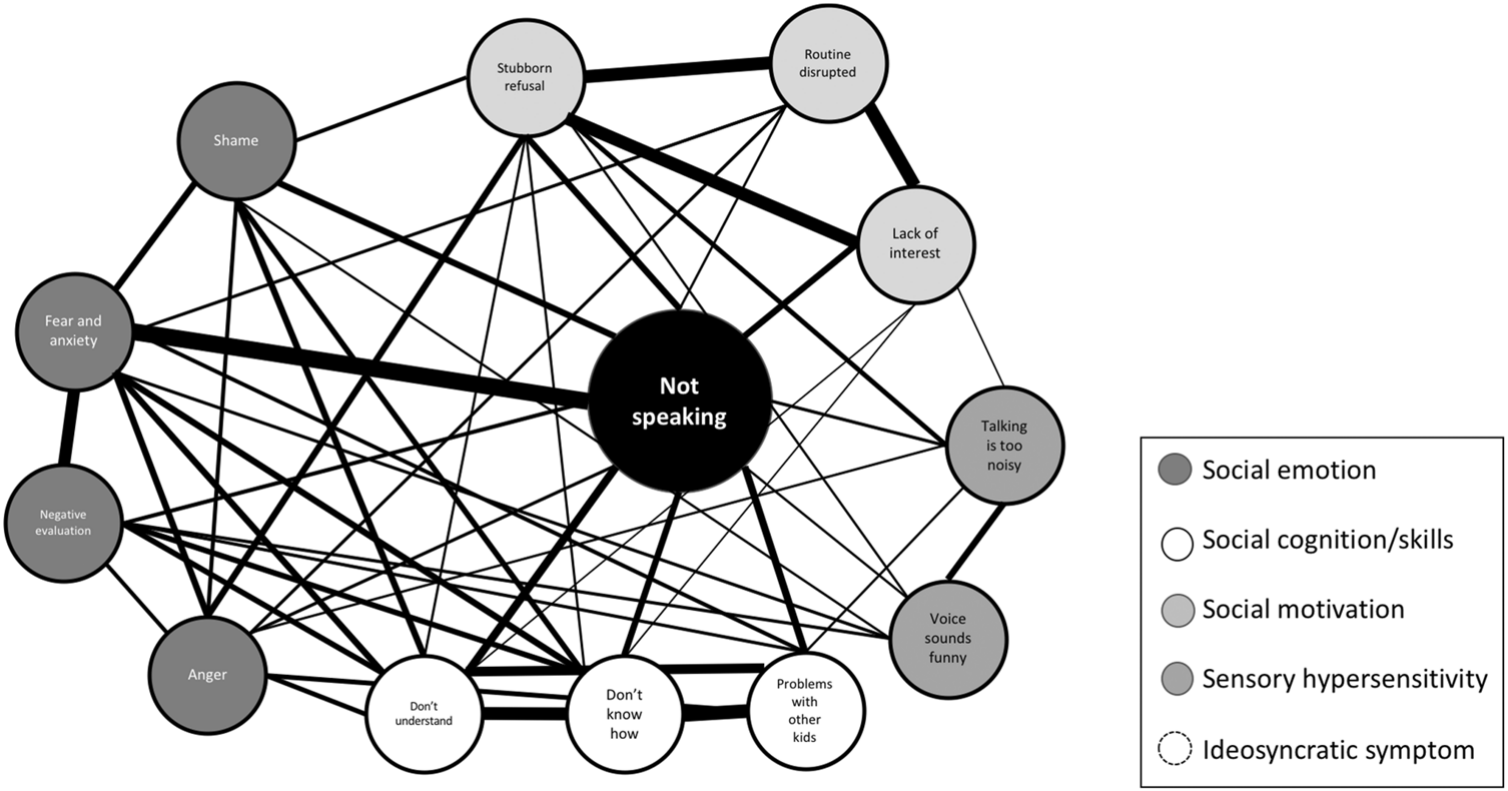
Possible network structure of SM including anxiety and ASD-related symptoms. Not speaking is the key symptom of the disorder that is related to other symptoms reflecting social emotion, social cognition/skills, social motivation, and sensory hypersensitivity. Lines reflect the hypothetical associations among symptoms, with thicker lines indicating stronger associations

**Fig. 3:**
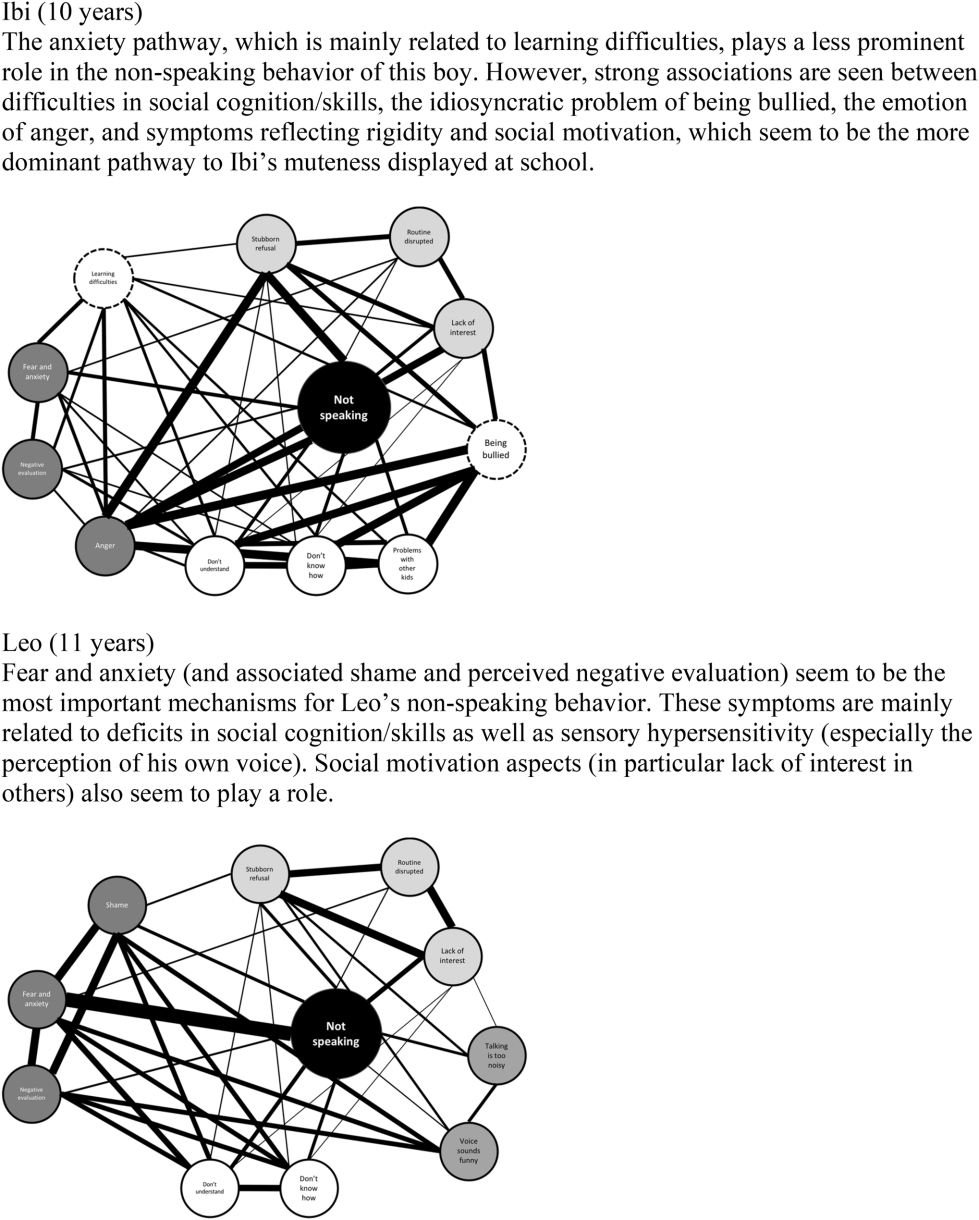
Illustrations of the network structure applied to two cases of children with SM (note that these models are merely hypothetical: they are not based on empirical data but are construed on information obtained during anamnesis and diagnostic assessment)

ASD is accompanied by a range of social difficulties as well as certain peculiarities (RRBIs) that likely fuel children’s feelings of discomfort and apprehension of social situations, thereby making a contribution to the persistent non-speaking behavior that is so characteristic for SM. Note that this observation fits nicely with the network approach to psychopathology (Borsboom and Cramer 2013), which assumes that disorders consist of a network of symptoms that interact in ways that tend to maintain themselves. As illustrated in Fig. 2, the persistent and repeatedly occurring non-speaking behavior of a child with SM can be viewed as the central symptom in a network of functionally related symptoms. Note that the network approach also allows for heterogeneity within the disorder. When applying this model hypothetically to the cases of Ibi and Leo that were presented at the start of this review, it becomes clear that there are a number of common features but also various differences in the clinical presentation of both boys (Fig. 3). Future research should make an attempt to further explore the proposed symptom network of children with SM as this could not only bolster the role of anxiety but also yield valuable information on the contribution of ASD-related (as well as other) symptoms to this psychiatric condition (cf. Montazeri et al. 2019).

### Clinical Implications for Assessment

An important clinical implication of the observation that ASD is linked to SM is of course that the presence of (symptoms of) this neurodevelopmental disorder needs to be assessed during the diagnostic evaluation of children who do not speak in specific situations. We think this is a necessary procedure that will give clinicians more insight in the pathogenesis of the non-speaking behavior of a child and will help them make an informed decision about treatment. The socio-emotional consequences of SM are severe enough to justify the effort and associated costs, and this approach also seems feasible given the rarity of the psychiatric condition. However, as ASD will not be present in every child with SM, one could adopt the two-step procedure as proposed by Volkmar et al. (2014) to conduct such an assessment effectively and economically. This procedure entails a first screening of ASD symptoms by means of a scale that has been specifically developed for this purpose, such as the Autism-Spectrum Quotient (Baron-Cohen et al. 2001a, b), the Social Communication Questionnaire (Rutter et al. 2003), or the aforementioned SRS (Constantino and Gruber 2005, or in case these are not available one could even employ a selective set of items taken from the BASC (Reynolds and Kamphaus 2015) or the widely used Achenbach (2009) scales (see Deckers et al. 2020). If the initial screening indicates significant symptomatology, a more thorough and indepth diagnostic evaluation could be conducted using the aforementioned ADOS and ADI to determine the presence and severity of ASD. Thus, apart from standard procedures to assess the language/speech, cognitive, and medical (e.g., audiological) functioning of children with SM (Dow et al. 1995), the assessment should entail a detailed psychiatric evaluation, which includes an assessment of (social) anxiety symptoms as well as an evaluation of possibly present ASD symptomatology.

### Implications for Treatment

The proper assessment of ASD symptomatology is also highly relevant for the treatment of children who do not speak in specific social situations. In case a child appears to display no clear signs of ASD, it is most appropriate to treat SM as an anxiety disorder. As such, psychological interventions should be primarily cognitive-behavioral in nature (see Farrell et al. 2019), meaning that exposure to verbal communication in feared situations as well as restructuring threat-related, negative thinking into more positive thinking need to be important elements of treatment. In the literature, only a number of randomized controlled trials (RCTs) can be found that examined whether the cognitive-behavioral approach is indeed an effective intervention for children with SM. An exemplary study was conducted by Bergman et al. (2013) who evaluated the efficacy of integrated behavioral therapy (IBT), a 20-session protocol consisting of gradual exposure to speech-related situations, which was supported by behavioral techniques such as reinforcement, shaping, and modeling. Dependent on children’s developmental level, cognitive restructuring was also conducted targeting the replacement of anxious thoughts with coping self-statements. Twenty-one children with SM aged 4 to 8 years were randomly assigned to either the IBT protocol or a waiting list, and treatment outcome was evaluated in terms of diagnostic status, symptom levels on standardized parent- and teacher-report questionnaires, and speech production during behavioral tasks. The results showed that 67% of the children treated with the IBT protocol no longer fulfilled the diagnostic criteria of SM. Further, pre- to post-treatment comparisons revealed that symptoms of SM and social anxiety as reported by parents and teachers were significantly reduced whereas no such decrease was found in the waiting list condition. Finally, on the behavioral speech tasks, children who had been treated with the protocol employed on average three times as many words as compared to before the intervention, while the number of words used in the control group did not change from the pre- to post-assessment. Similar positive results have been obtained in RCTs conducted by Oerbeck et al. (2014) and Cornacchio et al. (2019), leading to the conclusion that cognitive-behavioral interventions are indeed effective for many children with SM (Zakszeski and DuPaul 2017).

Cognitive-behavioral interventions have proven to be an efficacious treatment for children with anxiety disorders in general (Reynolds et al. 2012), but another viable treatment option for children with anxiety problems is pharmacotherapy (Muris 2012; Ollendick and March 2004). In particular, selective serotonin reuptake inhibitors (SSRIs) have been shown to be useful in this regard: this type of medication is relatively safe to use, has limited side effects, and—most importantly—has been shown to produce clinically significant symptom reductions in children with anxiety disorders (e.g., Walkup et al. 2008). Support for the use of SSRIs in children with SM is limited, however. A controlled study was conducted by Black and Uhde (1994) who treated 16 children with SM (aged 5–16 years) with placebo medication for 2 weeks. The 15 non-responders were then randomly assigned to a double-blind treatment with either fluoxetine or continued placebo for an additional 12 weeks. The results showed that all children showed significant improvements in symptoms at the post-treatment assessment, but those who had received fluoxetine displayed significantly greater improvements in SM symptoms and global functioning as rated by the parents than the placebo-treated controls. Other studies on the effects of SSRIs in children with SM have been conducted, but these mainly involve open trails or case reports (e.g., Kaakeh and Stumpf 2008). So, given the current status of research, it seems most appropriate that SM is first treated with a cognitive-behavioral intervention, whereas pharmacotherapy with SSRIs could be added in case such an approach yields no or insufficient results (Ostergaard 2018).

In case ASD (symptoms) are present in children with SM, interventions targeting the (social) anxiety symptoms are still applicable, which means that cognitive-behavioral therapy and perhaps supplemented by pharmacotherapy with SSRIs remain the main spearpoints in treatment (Thorkelson et al. 2019; Wood et al. 2009). However, it is important to note that various scholars have noted that these interventions are less effective in children with ASD. For example, in a meta-analysis exploring complete recovery rates of children with anxiety disorders who had been treated with cognitive behavior therapy, Warwick et al. (2017) noted that approximately 66% no longer met the diagnostic criteria of any anxiety disorder at the end of the intervention. However, there was considerable heterogeneity in the full recovery rates across these studies, and additional analysis revealed that the presence of ASD was an important moderator of outcome. More precisely, the data showed that whereas in typically developing children 61% no longer fulfilled the criteria of any anxiety disorder, the percentage of such full recovery was only 23% in children with ASD. Thus, although researchers have made an effort to optimize the delivery of cognitive behavioral treatment in children with ASD (e.g., by increased use of visual aids, providing more structure, incorporating more and longer sessions, and adding more relaxation exercises; Chalfant et al. 2007), the efficacy of this intervention appears more limited in this population.

A similar conclusion has been reached with regard to the treatment of anxiety disorders in children with ASD by means of SSRIs. Williams et al. (2013) conducted a detailed review investigating the effects of SSRIs on various aspects of functioning in individuals with ASD and noted that “there is no evidence that selective serotonin reuptake inhibitors (SSRIs) are effective as a treatment for children with autism” (p. 16). However, it needs to be mentioned that the limited number of studies, in particular on the effects of SSRIs on anxiety, prevents a solid and valid interpretation. Obviously, this is a topic for further scientific inquiry. Meanwhile, it is not that surprising that children with ASD respond less well to the standard treatment offered for anxiety disorders. As we have seen in the current review, the social difficulties of children with this neurodevelopmental disorder go further than dysregulated social emotion (which includes subjective feelings of fear and anxiety, physiological symptoms, and avoidance behavior), but also encompass marked problems in social cognition, social skills, and social motivation as well as other detrimental effects related to the presence of RRBIs. Further, at the brain level, ASD is a complex type of psychopathology involving multiple neurocircuits and associated neurotransmitter systems (Frith 2003).

All this implies that interventions for individuals with ASD may need to include a greater variety of components that target different aspects of social functioning (Pallathra et al. 2019). Fortunately, a wide range of interventions are available that can be used for this purpose. For example, group-based trainings have been developed to improve the social skills of children with ASD, and in general research (including a good number of RCTs) has demonstrated that such interventions are reasonably effective (Gates et al. 2017; Reichow and Volkmar 2010). Furthermore, programs addressing the prototypical emotion recognition and theory of mind difficulties displayed by children with ASD can also be found. One example is “Mind Reading”, a computerized intervention to increase children’s abilities to understand the emotions of other people and to engage in appropriate social behavior (LaCava et al. 2010), which has shown promising results in children with ASD aged 6 years and older (Kouo and Egel 2016). In addition, programs are beginning to emerge that aim to improve the social motivation of children with ASD. Noteworthy in this regard is the Pivotal Response Intervention for Social Motivation (Vernon 2014), a behavioral modification program that systematically couples specific social cues offered to elicit a child’s response with a motivating, rewarding stimulus (usually a highly preferred toy or object). The initial experiences with this type of treatment have been positive but need to be tested in more large-scale studies (Vernon et al. 2019). Finally, even for RRBIs—which as noted earlier are thought to play a role in the perseverance of social difficulties of children with ASD, specific treatment strategies have been developed (Boyd et al. 2012). For instance, to break insistence on sameness and rigidity, Miller and Neuringer (2000) have described a behavioral technique that involves reinforcing children for varying their behavioral responses, with the strength of reinforcement being dependent on how novel the displayed behavior is. In the case of sensory hypersensitivity, procedures can be employed that either accommodate sensory difficulties—for example, by using headphones to attenuate noise in crowded social situations (Pfeiffer et al. 2019), or interventions that actually reduce the hyper-reactivity to sensory input, such as a systematic desensitization procedure guided by a hierarchy of stimuli to which the child is oversensitive (Koegel et al. 2004; see Muskett et al. 2019).

Apart from these psychological interventions—which are all based on learning principles and thus behavioral in nature, pharmacological interventions may also be helpful to reduce the prototypical symptomatology of children with ASD. While the efficacy of a wide range of psychiatric drugs have been evaluated in this population, the most promising evidence has been documented for antipsychotics such as aripiprazole and risperidone (Siegel and Beaulieu 2012). Although their neural working mechanism is quite different, both types of medication have a positive effect on children’s social behavior and reduce the occurrence and/or severity of RRBIs. More precisely, various placebo-controlled studies have indicated that aripiprazole and risperidone reduce irritability in children with ASD (Marcus et al. 2009; Owen et al. 2009; Research Units on Pediatric Psychopharmacology Autism Network 2002), and most importantly significantly improved their adaptive skills as well as social functioning (Varni et al. 2012; Williams et al. 2006). Although the effects of antipsychotic agents such as aripiprazole and risperidone should not be overstated (e.g., Marrus et al. 2014), they can be helpful in the management of the extremely difficult behaviors of children with ASD and the mitigation of associated social difficulties.

Taken together, the observation that SM sometimes is a psychiatric condition at the crossroads of SAD and ASD has clear implications for the treatment of this disorder. That is, in those cases for which a careful psychodiagnostic evaluation has shown that SM is not merely an anxiety disorder but ASD (symptomatology) is implicated as well, clinicians need to provide appropriate psycho-education, set realistic treatment goals, and temper expectations regarding outcome towards parents as ASD generally is a more difficult-to-treat and persistent psychiatric problem. They should consider to not only deploy interventions that target dysregulated emotional responses (in particular fear and anxiety) in social situations, but also have an eye for the other prototypical difficulties in social functioning of children with this neurodevelopmental disorder including deficits in social skills, social cognition, and social motivation, which need to be addressed in treatment as well. This means that besides cognitive-behavior therapy and eventually SSRIs, other training programs aiming to improve social skills, emotion recognition, mind reading abilities, and social interest as well as anti-psychotic medication should be considered as additional treatment options (Volkmar et al. 2014).

### Implications for Research on SM

So far, the evidence for the relationship between SM and ASD is based on clinical reports (e.g., Holka-Pokorska et al. 2018) and a restricted number of empirical studies that have been conducted by researchers who went beyond the current classification criteria to explore the co-occurrence (Klein et al. 2019; Steffenburg et al. 2018) and/or hypothesized shared biological or psychological features of both disorders (Cholemkery et al. 2014; Stein et al. 2011; Nowakowski et al. 2011). Obviously, we need more clinical studies as well as large-scale epidemiological research to get a better picture of the actual comorbidity of these disorders. Given that psychiatric problems are no longer perceived as categorical entities but more as dimensional phenomena, another important next step in the research would be to further explore the link between SM (and social anxiety) and ASD symptoms in typically developing children (Muris 2020). Such studies could also include an assessment of other mental health and developmental issues that have been related to SM in the extant literature. This would give a picture of the relative contributions of social anxiety, ASD, and other problems to SM.

Another possibility would be to go beyond the symptom level and to map the social difficulties of children with SM in more detail. So far, studies on this topic have been quite sparse, but early studies by Nowakowski et al. (2011)—who explored impairments in joint attention—and Cunningham et al. (2006)—who investigated the social skills of children with SM—are good examples of such an approach. Further investigations could extend this knowledge and also focus on other aspects of social functioning such as mind reading and emotion recognition abilities and social interest and motivation. Another viable route for research could be to examine possible transdiagnostic processes that may underlay the social difficulties of children with SM and its psychiatric allies of SAD and ASD, including inhibited temperament characteristics (Gensthaler et al. 2016a; Muris et al. 2016), emotion regulation impairments (Weissman et al. 2019; White et al. 2014a, b), and dysregulations in ‘social’ brain circuitry (Caouette and Guyer 2014; Kennedy and Adolphs 2012).

In the past decades, a number of high-quality studies have been conducted on the treatment of SM by adopting a cognitive-behavioral approach (Bergman et al. 2013; Cornacchio et al. 2019; Oerbeck et al. 2014). In general, this type of intervention has been shown to be reasonably effective, but it would be worthwhile to study whether efficacy levels can be raised by adding treatment components that are currently specifically used in ASD children (e.g., interventions to improve theory of mind, social motivation etc.). Similarly, in the case of the pharmacological treatment of SM, research has primarily focused on SSRIs, but it might be worthwhile to also explore the clinical potential of anti-psychotic medication such as aripiprazole (Ipci et al. 2017), although it needs to be stressed that such a treatment approach is only indicated when the presence of ASD symptomatology has been clearly established.

## Conclusion

In the latest versions of classification systems such as the DSM (American Psychiatric Association 2013) and ICD (World Health Organization 2018), SM is categorized as an anxiety disorder. As noted throughout this review, this is justified when looking at the abundant empirical evidence demonstrating that (social) anxiety is a common symptom associated with this psychiatric condition (Driessen et al. 2020; Cohan et al. 2008; Diliberto and Kearney 2016, 2018; Schwenck et al. 2019; Vogel et al. 2019). The acknowledgement of SM being an anxiety problem is strongly guiding our thinking about the origins of the disorder, which is reflected in developmental psychopathology models that are dominated by anxiety-relevant etiological factors (Cohan et al. 2006; Muris and Ollendick 2015; Viana et al. 2009). In line with this, current treatments of SM can be summarized under the header of “Reinforce, shape, expose, and fade” (Zakszeski and DuPaul 2017, p. 1), indicating that (cognitive) behavioral approaches, just like in other anxiety disorders (e.g., Rapee et al. 2009), are the dominant intervention.

It is not our intention to downplay the role of (social) anxiety in SM, but rather we want to point out that SM may also be connected to ASD and that this may have important implications for the clinical management as well as research of children with this disorder. More precisely, we have argued that there seems to be a group of children with SM in which the key symptom of muteness is not entirely based on fear and anxiety, but ASD symptomatology plays a prominent role as well. For clinicians, it is important to have an eye out for these children as their treatment might require not only to focus on the alleviation of anxiety but also to target other prototypical social difficulties displayed by children on the autism spectrum. This was also true for the cases of Ibi and Leo described at the beginning of this article: for both boys, we started energetic with an anxiety-focused cognitive-behavioral approach, but after some time realized that we had to take a step back and after an appropriate assessment adjusted our intervention plan, making it more appropriate for these children on the crossroads of SAD and ASD.

## Footnotes

1 In Leo this mainly occurred when he was upset due to a (for him) distressing situation, but in Ibi these problems mainly occurred at home when he was asked to do something or when his actions were corrected, displaying more behavior of an oppositional-defiant nature. The two boys were also different in terms of intellectual functioning, with Ibi having an estimated total IQ of 81 (assessed by means of the revised Snijders-Oomen Non-Verbal Intelligence Test; Tellegen & Laros, 1993) and receiving special needs education, and Leo displaying a total IQ of 126 (with a large discrepancy between the verbal and non-verbal scores, respectively 103 versus 144, measured with the Wechsler Intelligence Scale for Children; Wechsler, 1991) and still attending a regular primary school. Further, Leo appeared to a have a hypersensitivity for sounds (including a dislike of his own voice), whereas Ibi did not show such a sensitivity and sometimes at home could produce a blast of noise.
